# Novel Cre-Expressing Mouse Strains Permitting to Selectively Track and Edit Type 1 Conventional Dendritic Cells Facilitate Disentangling Their Complexity *in vivo*

**DOI:** 10.3389/fimmu.2018.02805

**Published:** 2018-12-04

**Authors:** Raphaël Mattiuz, Christian Wohn, Sonia Ghilas, Marc Ambrosini, Yannick O. Alexandre, Cindy Sanchez, Anissa Fries, Thien-Phong Vu Manh, Bernard Malissen, Marc Dalod, Karine Crozat

**Affiliations:** ^1^Centre d'Immunologie de Marseille-Luminy, Turing Center for Living Systems, CNRS, INSERM, Aix Marseille Univ, Marseille, France; ^2^Centre d'Immunophénomique, Aix Marseille Univ, CNRS, INSERM, Marseille, France

**Keywords:** dendritic cells, cDC1, XCR1, Gp141b, Karma, Clec9a, Cre, fate mapping

## Abstract

Type 1 conventional DCs (cDC1) excel in the cross-priming of CD8^+^ T cells, which is crucial for orchestrating efficient immune responses against viruses or tumors. However, our understanding of their physiological functions and molecular regulation has been limited by the lack of proper mutant mouse models allowing their conditional genetic targeting. Because the *Xcr1* and *A530099j19rik* (*Karma/Gpr141b)* genes belong to the core transcriptomic fingerprint of mouse cDC1, we used them to engineer two novel Cre-driver lines, the *Xcr1*^*Cre*^ and *Karma*^*Cre*^ mice, by knocking in an IRES-Cre expression cassette into their 3′-UTR. We used genetic tracing to characterize the specificity and efficiency of these new models in several lymphoid and non-lymphoid tissues, and compared them to the *Clec9a*^*Cre*^ mouse model, which targets the immediate precursors of cDCs. Amongst the three Cre-driver mouse models examined, the *Xcr1*^*Cre*^ model was the most efficient and specific for the fate mapping of all cDC1, regardless of the tissues examined. The *Karma*^*Cre*^ model was rather specific for cDC1 when compared with the *Clec9a*^*Cre*^ mouse, but less efficient than the *Xcr1*^*Cre*^ model. Unexpectedly, the *Xcr1*^*Cre*^ model targeted a small fraction of CD4^+^ T cells, and the *Karma*^*Cre*^ model a significant proportion of mast cells in the skin. Importantly, the targeting specificity of these two mouse models was not changed upon inflammation. A high frequency of germline recombination was observed solely in the *Xcr1*^*Cre*^ mouse model when both the *Cre* and the *floxed* alleles were brought by the same gamete irrespective of its gender. *Xcr1, Karma*, and *Clec9a* being differentially expressed within the cDC1 population, the three CRE-driver lines examined showed distinct recombination patterns in cDC1 phenotypic subsets. This advances our understanding of cDC1 subset heterogeneity and the differentiation trajectory of these cells. Therefore, to the best of our knowledge, upon informed use, the *Xcr1*^*Cre*^ and *Karma*^*Cre*^ mouse models represent the best tools currently reported to specifically and faithfully target cDC1 *in vivo*, both at steady state and upon inflammation. Future use of these mutant mouse models will undoubtedly boost our understanding of the biology of cDC1.

## Introduction

Dendritic cells (DCs) constitute a heterogeneous population of antigen presenting cells (APCs) which are instrumental for the orchestration of innate and adaptive immune responses. In mice and in humans, three distinct types of DCs differing in their phenotype, localization and functions populate all lymphoid and most non-lymphoid tissues at steady state. Plasmacytoid DCs (pDCs) are the major source of type I interferon (IFN) upon many viral infections. Conventional DCs (cDCs) consist of two populations, coined as type 1 and type 2 cDCs and which excel in the cross-priming of CD8^+^ T cells or in the promotion of CD4^+^ T cell and humoral immunity, respectively. The functions of cDCs and their molecular regulation have been studied *in vivo* by using a wealth of mouse models that enable their depletion or genetic manipulation, namely *Cd11c (Itgax)*^*hDTR*^ (1) or *Cd11c (Itgax)*^*Cre*^ (2, 3) and more recently the *Zbtb46*^*hDTR*^ (4) or *Zbtb46*^*Cre*^ (5). However, interpretation of the results obtained using those mice can be difficult due to the expression of *Cd11c* by many other cell types than cDCs and of *Zbtb46* by committed erythroid progenitors and endothelial cell populations (6). Moreover, these mutant mouse models are not suited to study the respective functions of each of the two cDC types. This goal requires the use of refined mutant mouse models enabling specific targeting of either cDC1 or cDC2.

Constitutive (*Batf3*-KO mice) or conditional (*Itgax*^*Cre*^; *Irf8*^*fl*/*fl*^ mice) genetic inactivation of transcription factors required for the differentiation of cDC1 allowed to study their specific functions *in vivo* (7, 8). However, interpretation of the results obtained with these models can be difficult because they are not targeting solely cDC1 (7, 9–11). Moreover, cDC1 are replenished in *Batf3*-KO mice under inflammatory conditions, due to expression of other Batf transcription factors that compensate for Batf3 loss (12). Finally, these models do not allow the editing of cDC1 genome, which would be a powerful method to decipher the molecular regulation of their functions. Hence, novel mutant mouse models are needed to reach this goal.

In all tissues with the exception of the intestine, cDC1 can be defined as CD24^+^ SIRPα/CD172a^−^ cDCs (13–15). In addition, lymphoid-tissue resident cDC1 express CD8α, whereas the cDC1 residing in the parenchyma of non-lymphoid tissues and their counterparts that have migrated in secondary lymphoid organs express CD103. CLEC9A, a C type lectin receptor that allows efficient cross-presentation by cDC1 of dying cell-associated antigens (16) has been identified as a good candidate to generate mice enabling selective targeting of cDC1 *in vivo* due to its selective expression in these cells and to a lesser extent in pDCs (17–20). However, a thorough analysis of mice expressing a Cre recombinase under the *Clec9a* promoter showed that Cre-driven recombination occurred not only in cDC1 and to some extent in pDCs, but also in cDC2, leading to the discovery that *Clec9a* is expressed in a progenitor cell common to both cDC types (21). Hence, the *Clec9a*^*Cre*^ mouse is not suitable for specific targeting of cDC1.

A major breakthrough in the field of cDC1 was the identification of XCR1 as a universal marker of all cDC1 regardless of their tissues of residency, and present in all the warm-blooded vertebrate species studied to date (22–27). *Xcr1* encodes the chemokine receptor XCR1, which ligand XCL1 is strongly upregulated in natural killer (NK) cells, CD8^+^ T cells and memory T cells upon activation in mice (24, 26, 28–31). Recently, a mouse model based on the expression of the Cre recombinase under the control of the *Xcr1* promoter has been generated to specifically manipulate gene expression in cDC1. This mutant mouse model was engineered by replacing the single coding exon of *Xcr1* by the *Cre* gene (32). This strategy assumes that the *Xcr1* gene is haplosufficient. However, this hypothesis has to be tested considering that XCR1 promotes the cross-talk between cDC1 and NK cells or CD8^+^ T lymphocytes, by facilitating their reciprocal recruitment and/or activation (24, 26, 29). Regardless of its potential limitation, this *Xcr1*^*tm*4(*cre*)*Ksho*^ mouse model has been useful to decipher the role of cDC1 in intraepithelial T cell homeostasis in the intestine (32). However, to the best of our knowledge, it has not been used yet for conditional gene targeting of the cDC1 lineage. Besides *Xcr1*, the *A530099j19rik* gene (named *Karma* hereafter) has also been identified as selectively expressed in cDC1 by bulk transcriptomic analysis on immune cell subsets and organs (10, 33). The *Karma* gene encodes a protein with 7 transmembrane domains, likely corresponding to a G protein-coupled receptor, leading to its recent denomination as *Gpr141b* by the Mouse Genome Informatics. Recently, we generated the *Karma* knock-in reporter/deleter mouse model, which expresses in the *Karma* locus a construct encoding both the fluorescent tandem dimer Tomato (tdTomato) and the human diphtheria toxin receptor (hDTR), allowing specific tracking and conditional depletion of cDC1 *in vivo*. Results obtained with this reporter mouse validated the *Karma* locus as highly reliable to functionally target cDC1 *in vivo* (33).

To match the unmet need of a mouse model allowing specific and efficient *in vivo* genetic manipulation of cDC1, we generated two novel Cre-driver lines, the *Xcr1*^*Cre*^ and *Karma*^*Cre*^ mice, by knocking in an IRES-Cre expression cassette in the 3′-UTR of the *Xcr1* or *Karma* locus, respectively. In this study, we used genetic tracing to characterize the specificity and efficiency of the Cre-mediated recombination in these new models at steady state and upon infection, and compared them with the *Clec9a*^*Cre*^ model. This study also advanced our understanding of the phenotypic heterogeneity of cDC1 with regard to their differentiation trajectory.

## Materials and Methods

### Generation of cDC1 Targeting Cre Constructs and Mice

*Xcr1*^*Cre*^ (*B6-Xcr1*^*tm*1*Ciphe*^) and *Karma*^*Cre*^ (*B6-Gpr141b*^*tm*2*Ciphe*^) mice were made according to a standard gene targeting approach in C57BL/6N-derived ES cells. They were constructed by inserting, through ET homologous recombination, a cassette containing the internal ribosome entry site (IRES) followed by a gene encoding the codon-improved version of Cre recombinase (34), into the 3′-UTR of the *Xcr1* or *A530099j19rik/Gpr141b* genes, 34 and 98 bp after the stop codon, respectively. These mice were outcrossed for three generations with wild type (*Wt*) C57BL/6J mice purchased from Charles River Laboratories. All experiments were performed with sex-matched littermate mice at 6–12 weeks of age. *Clec9a*^*Cre*^ (*Clec9a*^*tm*2.1(*icre*)*Crs*^) (21) knock-in mice [kindly provided by Caetano Reis e Sousa (The Francis Crick Institute, UK)], *Karma-tdTomato-hDTR* (*Gp141b*^*tm*1*Ciphe*^) (33) were maintained on the C57BL/6J background. *Rosa26*^*lox*−*stop*−*lox*−*tdRFP*^ (*Gt(ROSA)26Sor*^*tm*1*Hjf*^) mice in which expression of the tandem dimer Red Fluorescent Protein (tdRFP) is driven through the deletion of a “lox–stop–lox” sequence (35) were purchased from the Jackson Laboratory and maintained on the C57BL/6J background. *Rosa26*^*lox*−*stop*−*lox*−*DTA*^ (*Gt(ROSA)26Sor*^*tm*1(*DTA*)*Lky*^) mice in which expression of active domain of the diphtheria toxin (DTA) is driven through the deletion of a “lox–stop–lox” sequence (36) were obtained from Prs. David Voehringer and Richard M. Locksley, and maintained on the C57BL/6J background. Mice were bred and maintained in our specific pathogen—free animal facility. This study was carried out in accordance with institutional guidelines and with protocols approved by the Comité National de Réflexion Ethique sur l'Expérimentation Animale #14.

### Preparation of Cell Suspension From Blood and Tissues, and Analysis by Flow Cytometry

Splenocytes were prepared by infusing spleens with an enzymatic cocktail made of Collagenase D (1 mg/ml) and DNase I (70 μg/ml, both Roche) in plain RPMI 1640, and further incubation for 25 min at 37°C. Ice cold EDTA (2 mM) was added for additional 5 min. Cells were filtered through a 70-μm nylon sieve, and exposed to 0.155 M NH_4_Cl, 10 mM KHCO_3_, 0.127 M EDTA to lyse red blood cells. Liver and lungs were minced in an enzymatic cocktail (1 mg/ml of Collagenase D and 70 μg/ml of DNase I), incubated for 25 min at 37°C. Ice-cold EDTA (2 mM) was added for additional 5 min, then digested tissues were filtered through a 70 μm nylon sieve (BD Falcon). Low-density cells were further enriched by centrifugation over a 1.069 g/ml density gradient (OptiPrep, Axis-Shield), washed and resuspended in PBS, EDTA 2 mM, 2% BSA, and red blood cells were lysed as detailed above. Cutaneous LNs (inguinal and axillar LNs) were cut into small pieces and digested for 25 min at 37°C with a mixture of type II collagenase (Worthington Biochemical) and DNase I (Sigma-Aldrich) in plain RPMI 1640. The resulting cell suspension was treated with 5 mM EDTA and filtered through a 70 μm nylon sieve (BD Falcon). For the skin, ears were split into a ventral and dorsal parts and incubated for 105 min at 37°C in RPMI containing 0.25 mg/ml Liberase TL (Roche Diagnostic Corp.) and 0.5 mg/ml DNase I (Sigma Aldrich). Digested tissue was homogenized using Medicons and Medimachine (Becton Dickinson) to obtain homogenous cell suspensions. For skin mast cells, we used a protocol recently described (37). To test the germline recombination in blood cells, peripheral blood mononuclear cells (PBMCs) were enriched by centrifugation over a 1.077 g/ml density gradient (Ficoll-Paque Plus, GE Healthcare), washed and resuspended in PBS, EDTA 2 mM, 2% BSA before staining. Staining of cells for flow cytometry started with a pre-incubation with 2.4G2 mAb to block unspecific binding to Fc-receptors. Staining with mAb (Supplementay Table S1) was then performed in PBS, 2% BSA, 2 mM EDTA for 25 min on ice. For exclusion of dead cells 4′,6-diamidino-2-phenylindole (DAPI, Thermo Fisher Scientific) was added 5 min before acquisition. Data were acquired on a LSRFortessa X-20 flow cytometer (BD Biosciences), and analyzed using FlowJo (Tree Star, Inc.).

### Bone Marrow-Derived DC Differentiation

FLT3-L-BMDCs were generated as described (38) with some modifications. BM cell suspensions were prepared and red blood cells were lysed as detailed in the previous section. After washing in complete RPMI 1640 medium, cells were cultured at 3 × 10^6^ cells/ml in 24 well plates, with 10% FBS, RPMI 1640 medium containing murine FLT3-L (in house supernatant from B16-*Flt3l* cells, used at 1/20 final) at 37°C in 5% CO2. Four days after, half of the culture medium was replaced by fresh FLT3-L. Cells were harvested at indicated times for flow cytometry analysis after staining for CD11c, SiglecH, CD24, SIRPα, XCR1, and CD11b (Supplementay Table S1).

### Microarray Data Generation and Analysis

DCs were generated *in vitro* from mouse BM FLT3-L cultures and sorting by flow cytometry to over 98% purity, as live, singlet, CD11c^+^ cells that were SiglecH^+^ for eq-pDCs, SIRPα^−^CD24^high^ for eq-cDC1 and SIRPα^+^CD24^−/low^ for eq-cDC2. Total RNA (50 ng) was used as starting material for each sample to synthesize biotinylated probes, using the NuGen protocol as described previously (39). Affymetrix Mouse Gene 1.0 ST raw. CEL files were analyzed in the R statistical environment (version 3.4.1). Data were RMA normalized using the oligo package and processed as described previously (40). Heatmaps of Log2-normalized expression values of selected genes were performed using the Morpheus website from the Broad Institute (https://software.broadinstitute.org/morpheus/). Hierarchical clusterings were performed using the One-Pearson correlation as a metric and the average linkage as a clustering method for samples and genes, except for **Figure 5B** where the complete linkage method was used for the genes. The microarray data have been deposited in the GEO database under the series accession number GSE121859.

### Mouse Cytomegalovirus Infection

Animals were infected intraperitoneally with 2 × 10^5^ PFU of salivary gland-extracted MCMV Smith strain (3rd *in vivo* passage). Forty-Eight hours later, spleen and liver were harvested and prepared for flow cytometry analysis as described above.

### Analysis of Germline Recombination

Four females and 2 males of each *Xcr1*^*Cre*/*wt*^*; Rosa26*^*tdRFP*^^/wt^ and *Karma*^*Cre*/*wt*^*; Rosa26*^*tdRFP*^^/wt^ genotype were backcrossed to C57BL/6J mice. Their progeny was genotyped and bled to analyse tdRFP expression in circulating T and B cells as a sign of germline recombination.

## Results

### Generation of New cDC1-Targeting Cre-Driver Lines

**Figure 1:**
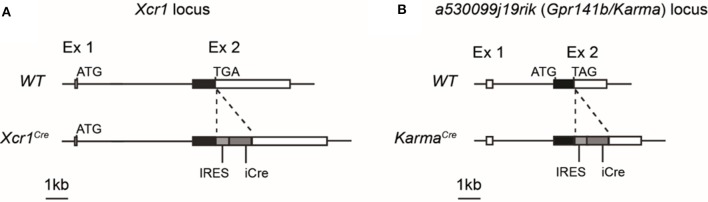
Schematic representations of *Xcr1*^*Cre*^ and *Karma*^*Cre*^ genetic constructions. A cassette containing an IRES sequence upstream of a gene encoding a codon-improved CRE recombinase was inserted by homologous ET recombination downstream of the stop codon of *Xcr1* exon 2 **(A)** and of *a530099j19rik/Karma/Gpr141b* exon 2 **(B)** genes, to produce *Xcr1*^*Cre*^ and *Karma*^*Cre*^ mouse mutants, respectively, on a C57BL/6J background.

By comparative gene expression profiling, we and other have previously identified *Xcr1* and *a530099j19rik* (*Gpr141b*/*Karma*) genes as specifically expressed by mouse cDC1 in different tissues throughout the body (10, 22–26, 31, 33). We used such unique gene expression profile to genetically target cDC1 *in vivo* by generating *Xcr1*^*Cre*^ (Figure 1A) and *Karma*^*Cre*^ (Figure 1B) knock-in mouse models. The insertion of an IRES-Cre cassette after the STOP codon of *Xcr1* and *Karma* genes allows the translation of two separate proteins resulting in the expression of the Cre recombinase. Expression of endogenous XCR1 was not significantly altered in the *Xcr1*^*Cre*^ mouse model (Supplementary Figure S1A, top).

### The *XCR1^*Cre*^* Mouse Model Allows Selective and Efficient Recombination of loxP Sequences in Migratory and Resident cDC1 in All Tissues Examined

**Figure 2:**
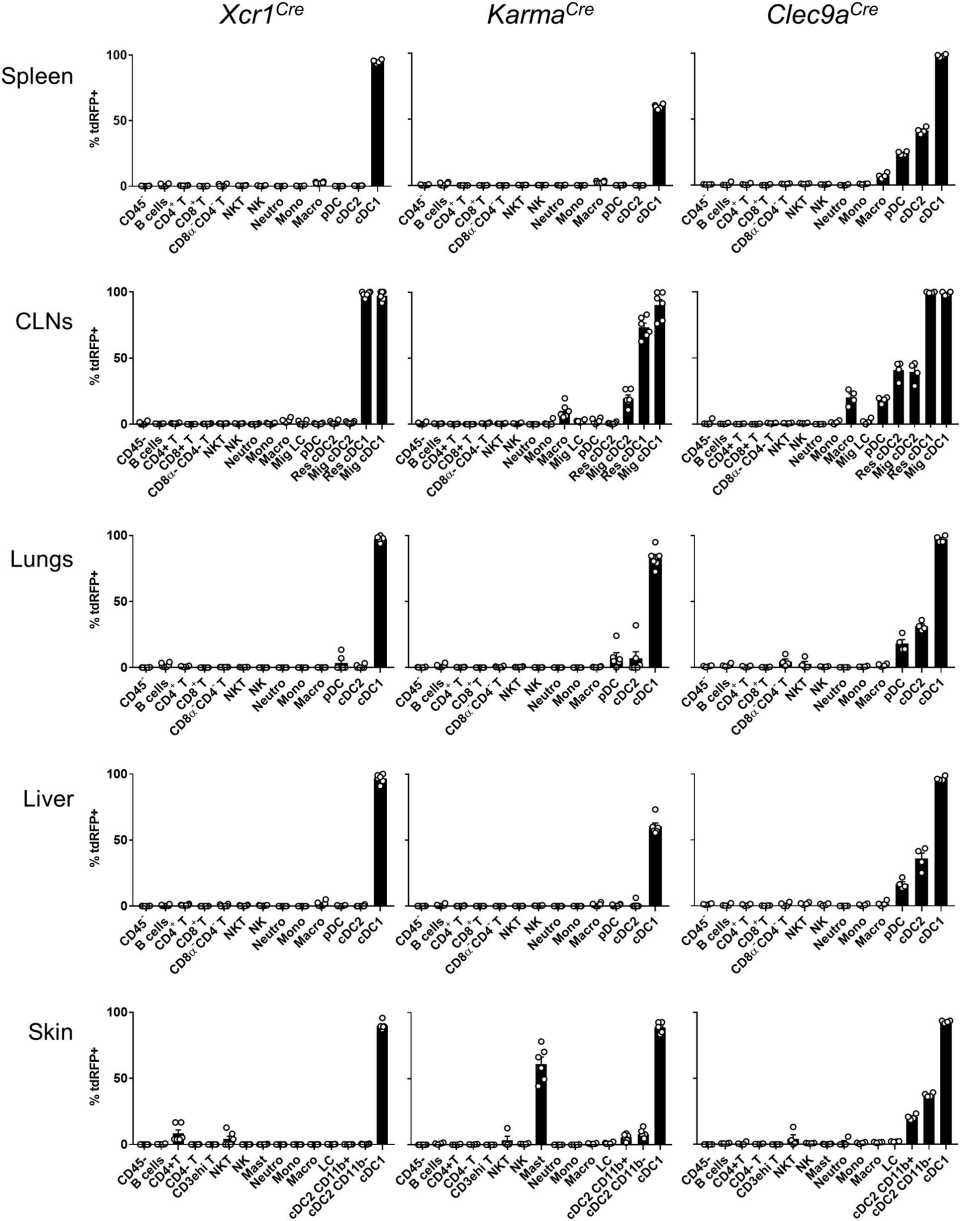
*Xcr1*^*Cre*^ and *Karma*^*Cre*^ mouse models target with high specificity cDC1 in lymphoid and non-lymphoid tissues. Flow cytometry analysis of the tdRFP expression by different immune cell populations (as defined in Supplementary Figure S1), from spleen, CLNs, lungs, liver, and skin of *Xcr1*^*Cre*/*wt*^*; Rosa26*^*tdRFP*^^/wt^, *Karma*^*Cre*/*wt*^*; Rosa26*^*tdRFP*^^/wt^ and *Clec9a*^*Cre*/*wt*^*; Rosa26*^*tdRFP*^^/wt^ mice. Gating strategies are detailed in Supplementary Figure S1. Data show one dot per individual value, mean +/– SEM per group, and are from two pooled experiments with at least two mice per group.

To determine the specificity of the Cre-induced recombination in *Xcr1*^*Cre*^ (Figure 1A) and *Karma*^*Cre*^ (Figure 1B) mice, we bred them with the Cre-reporter line *Rosa26*^*lox*−*stop*−*lox*−*tdRFP*^ (named hereafter *Rosa26*^*tdRFP*^) (35), and analyzed the tdRFP expression pattern in immune cells of lymphoid organs [spleen and cutaneous lymph-nodes (CLNs)] (Supplementary Figures S1A,B) and non-lymphoid tissues (lungs, liver, and skin) (Supplementary Figures S1C–E). To define DC cell populations, we applied gating strategies adapted from (41). As a control Cre-driver line, we used the *Clec9a*^*Cre*^ mice (21) bred to *Rosa26*^*tdRFP*^. Regardless of the tissues examined, we found that all three mouse models achieved effective targeting of the cDC1-lineage, with *Xcr1*^*Cre*^, and *Clec9a*^*Cre*^ being the most efficient (Figure 2). In contrary to *Clec9a*^*Cre*^, the *Xcr1*^*Cre*^ and *Karma*^*Cre*^ mouse models did not show any significant Cre activity, neither in pDCs nor in macrophages (Figure 2). However, Cre recombination (tdRFP signal) was detected in a fraction of other cell types. In *Xcr1*^*Cre*/*wt*^*; Rosa26*^*tdRFP*^^/wt^ mice, a minute proportion (< 1%) of CD4^+^ T cells expressed tdRFP in the spleen, CLNs, lung and liver, which increased to a much higher fraction of CD4^+^ T cells in the skin (8.4 ± 6.4%). The CD4^+^ T cells harboring *Xcr1*-driven Cre recombination lacked detectable level of XCR1 (Supplementary Figure S1E, bottom). Hence, they likely derived from progenitors cells that transiently expressed *Xcr1*. In the skin, lungs and CLNs of *Karma*^*Cre*/*wt*^*; Rosa26*^*tdRFP*^^/wt^ mice, a fraction of cDC2 had undergone recombination, although to a lesser extent than in *Clec9a*^*Cre*/*wt*^*; Rosa26*^*tdRFP*^^/wt^ mice where cDC2 were targeted in all tissues (Figure 2). Surprisingly, in the skin of *Karma*^*Cre*/*wt*^*; Rosa26*^*tdRFP*^^/wt^ mice, a large proportion of mast cells (61.1 ± 12.8%) also expressed the tdRFP (Figure 2).

**Figure 3:**
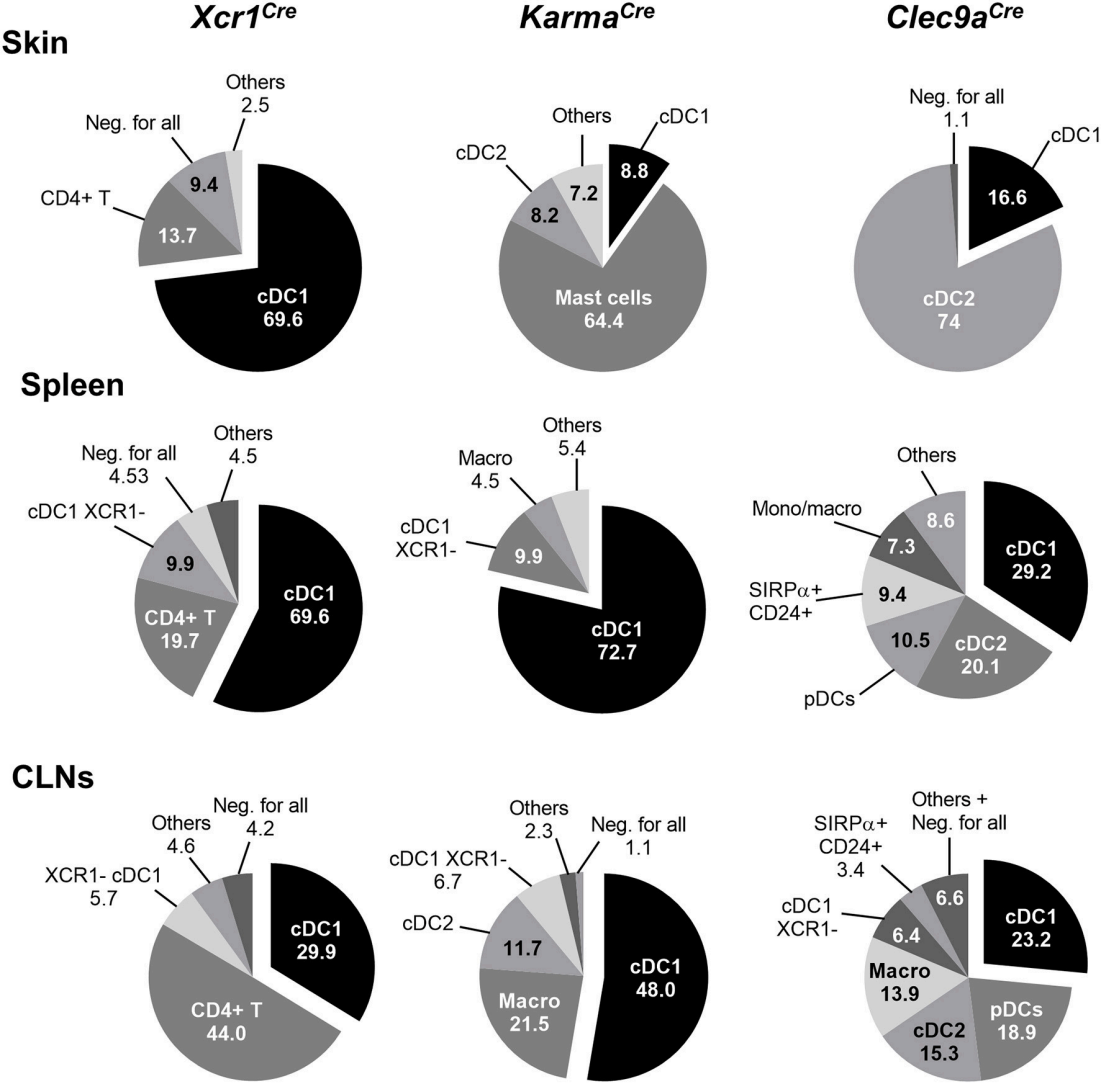
Proportion of cell types within tdRFP^+^ cells in the skin, spleen, and CLNs of each genotype. The back gating strategy used to define the relative proportions of cell types among tdRFP^+^ cells was performed by using the antibody staining detailed in the upper side of Supplementary Figure S1. Exceptions were made as follow for *Xcr1*^*Cre*/*wt*^*; Rosa26*^*tdRFP*^^/wt^ mice: to define further the tdRFP^+^ cells that fell within the Lin^+^ (CD19/CD3ε/NKp46/Ly6G) gate, the antibody staining detailed in the bottom side of Supplementary Figure S1 was used. The proportion of CD4^+^ T lymphocytes within tdRFP^+^ cells when using the latter (“lymphoid”) antibody panel was matching the proportion of Lin^+^ cells within tdRFP^+^ cells when using the former antibody (“myeloid”) panel. For *Karma*^*Cre*/*wt*^*; Rosa26*^*tdRFP*^^/wt^ mice, mast cells were defined as Lin^−^ CD11b^−^ CD11c^−^ XCR1^−^ MHC-II^−^ CD64^−^ F4/80^−^ FcεRIα^+^ CD117^+^. Others: sum of all the other cell subsets not detailed in the pie charts. Neg. for all, negative for all: cells that did not stain positive for the markers used in the upper antibody panel. Data are shown for one experiment representative of three with three mice per group.

To further assess cDC1-targeting specificity in the three Cre-driver mouse strains, we analyzed the proportion of different immune cell types within the tdRFP^+^ cells (Figure 3, Supplementary Figure S2). As expected on the basis of previous report (21), other cells than cDC1, in particular cDC2 and to some extent pDCs, represented the major fraction of the cells targeted in *Clec9a*^*Cre*^ mice (Figure 3). In contrast, in all examined organs, except for the CLNs, cDC1 represented the major fraction of the cells targeted in *Xcr1*^*Cre*^ mice, CD4^+^ T cells constituting the second most frequent cell types expressing tdRFP in these organs, and the most frequent in the CLNs. This reflects the higher numbers of CD4^+^ T cells as compared to cDC1s in all these organs. Finally, cDC1 represented the major fraction of targeted cells in *Karma*^*Cre*^ mice in all examined organs, except for the skin where 64.4 ± 5.8% of the tdRFP^+^ cells were mast cells (Figure 3). We assessed the expression pattern of the *Karma* gene in mast cells and compared it to a variety of other immune cell types, using the database from the Immgen consortium. These results revealed that mast cells express similar levels of the *Karma* gene as cDC1 in all organs examined, trachea, tongue, esophagus, skin, and peritoneal cavity (Supplementary Figure S3), consistent with the efficient genetic tracing of mast cells in the skin of *Karma*^*Cre*^ mice. Altogether, these results show that our novel *Xcr1*^*Cre*^ and *Karma*^*Cre*^ mouse models constitute the most reliable Cre-driver lines reported to date for selective and efficient *in vivo* targeting of cDC1, with the *Xcr1*^*Cre*^ model performing the best. However, it should be noted that a small fraction of CD4^+^ T cells is targeted in *Xcr1*^*Cr*^^e^ mice and that mast cells are largely targeted in *Karma*^*Cre*^ animals.

### The Use of Different cDC1-Specific Promoters for Driving Cre Expression Reveals Heterogeneity in the cDC1 Population Defined as CD24^+^ SIRPα^−^ cDCs

**Figure 4:**
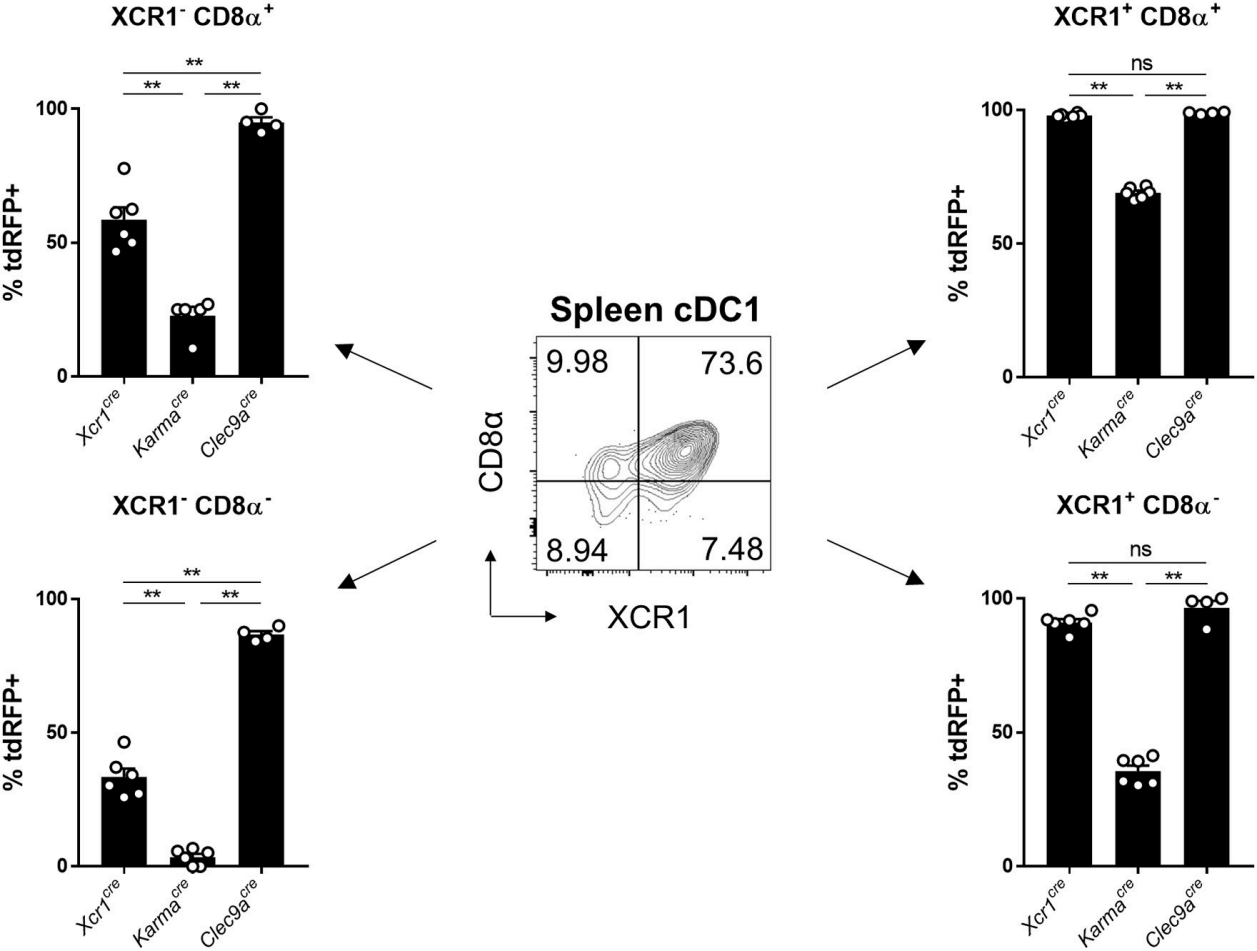
Tracing of *Xcr1* and *Karma* expression in the cDC1 population in spleen. Flow cytometry analysis of the tdRFP expression in the 4 CD24^+^ cDC1 subsets defined as CD8α^+^XCR1^−^, CD8α^+^XCR1^+^, CD8α^−^XCR1^+^, and CD8α^−^XCR1^−^, in the spleen of *Xcr1*^*Cre*/*wt*^*; Rosa26*^*tdRFP*^^/wt^, *Karma*^*Cre*/*wt*^*; Rosa26*^*tdRFP*^^/wt^, and *Clec9a*^*Cre*/*wt*^*; Rosa26*^*tdRFP*^^/wt^ mice. Data show one dot per individual value, mean +/- SEM per group, and are from two pooled experiments with at least two mice per group. Statistical analyses were performed using nonparametric Mann-Whitney tests in all experiments (^**^, *p* < 0.01; n.s, non-significant).

To try understanding the lower efficiency of the *Karma*^*Cre*^ model for cDC1 targeting, as compared to the *Xcr1*^*Cre*^ or *Clec9a*^*Cre*^ mice, we examined the expression pattern of the tdRFP reporter within the splenic cDC1 defined as CD24^+^SIRPα^−^ cDCs (gated in the CD45^+^Lin^−^SiglecH^−^MerTK^−^CD64^−^CD11c^+^MHC-II^+^CD26^+^ cells) (13). CD24^+^SIRPα^−^ cDC1 can be split into 4 subsets according to their heterogeneous expression of CD8α and XCR1 (Figure 4). XCR1 expression was reported to correlate with a better crosspresentation by cDC1 (23, 42). The CD8α^+^XCR1^+^ cDC1 subset was reported to be phenotypically and functionally homogenous (23, 42), and likely corresponds to full-fledged differentiated cDC1 endowed with a high crosspresentation activity (43). However, the three other cDC1 subsets, CD8α^+^XCR1^−^, CD8α^−^XCR1^+^, and CD8α^−^XCR1^−^, have not been extensively characterized. The CD8α^−^XCR1^−^ cells may encompass pre-cDC1 (13, 44). The CD8α^−^XCR1^+^ cells likely correspond to pre-terminally differentiated cDC1. The CD8α^+^XCR1^−^ cells could correspond to the small fraction of homeostatically matured splenic cDC1 that have downregulated XCR1 expression, similarly to what occurs at steady state in the skin, the intestine or the thymus (25, 32, 39). It could also be possible that XCR1^−^ cells encompass other cell types contaminating the SIRPα^−^CD24^+^ cDC1 gate. However, the exclusion of CD3ε^+^ and SiglecH^+^ cells in our gating strategy ensured that the CD8α^+^XCR1^−^ cDC1 subset was not contaminated by CD8^+^ T cells nor CD8α^+^ pDCs (45–47). Consistent with the early expression of *Clec9a* starting at the pre-DC stage (21), *Clec9a*^*Cre*^ model targeted efficiently all 4 subsets, regardless of XCR1 and CD8α acquisition, with more than 85% of the cells in each subset expressing tdRFP (Figure 4). Although the *Xcr1*^*Cre*^ model was more efficient in targeting XCR1^+^ cDC1 as initially expected, a significant Cre activity was also detected both in XCR1^−^CD8α^−^ and XCR1^−^CD8α^+^ cDC1 subsets, with 37% and 53% of tdRFP expression, respectively (Figure 4). This indicated that a significant proportion of these cells from these two subsets derived from XCR1-expressing precursors, consistent with the hypothesis that they respectively encompass pre-cDC1 and terminally matured cDC1. The *Karma*^*Cre*^ model was effective in targeting the XCR1^+^CD8α^+^ cDC1 subset, contrasting with no recombination detected in the XCR1^−^CD8α^−^ subset and with only a weak Cre activity in the XCR1^+^CD8α^−^ subset (Figure 4). The fraction of XCR1^−^CD8α^+^ cDC1 targeted in *Karma*^*Cre*^ mice (22.8 ± 6.1%) was lower than that of XCR1^+^CD8α^+^ cDC1 (69.0 ± 2.1%). This suggest that most of XCR1^−^CD8α^+^ cDC1 do not derive from XCR1^+^CD8α^+^ cDC1 contrary to our expectation that the majority of the former cells correspond to an advanced maturation state of the latter ones. Altogether, the combined use of our fate mapping mouse models suggests consecutive expression of the corresponding genes along the differentiation of the cDC1 lineage in the spleen, with *Clec9a* expressed from the common cDC progenitor stage, *Xcr1* likely starting at the pre-cDC1 stage and *Karma* turned on only at a later stage similarly to CD8α.

### Comparison of the Three Fate Mapping Mouse Strains Advances Our Understanding of the Differentiation Trajectory of cDC1

**Figure 5:**
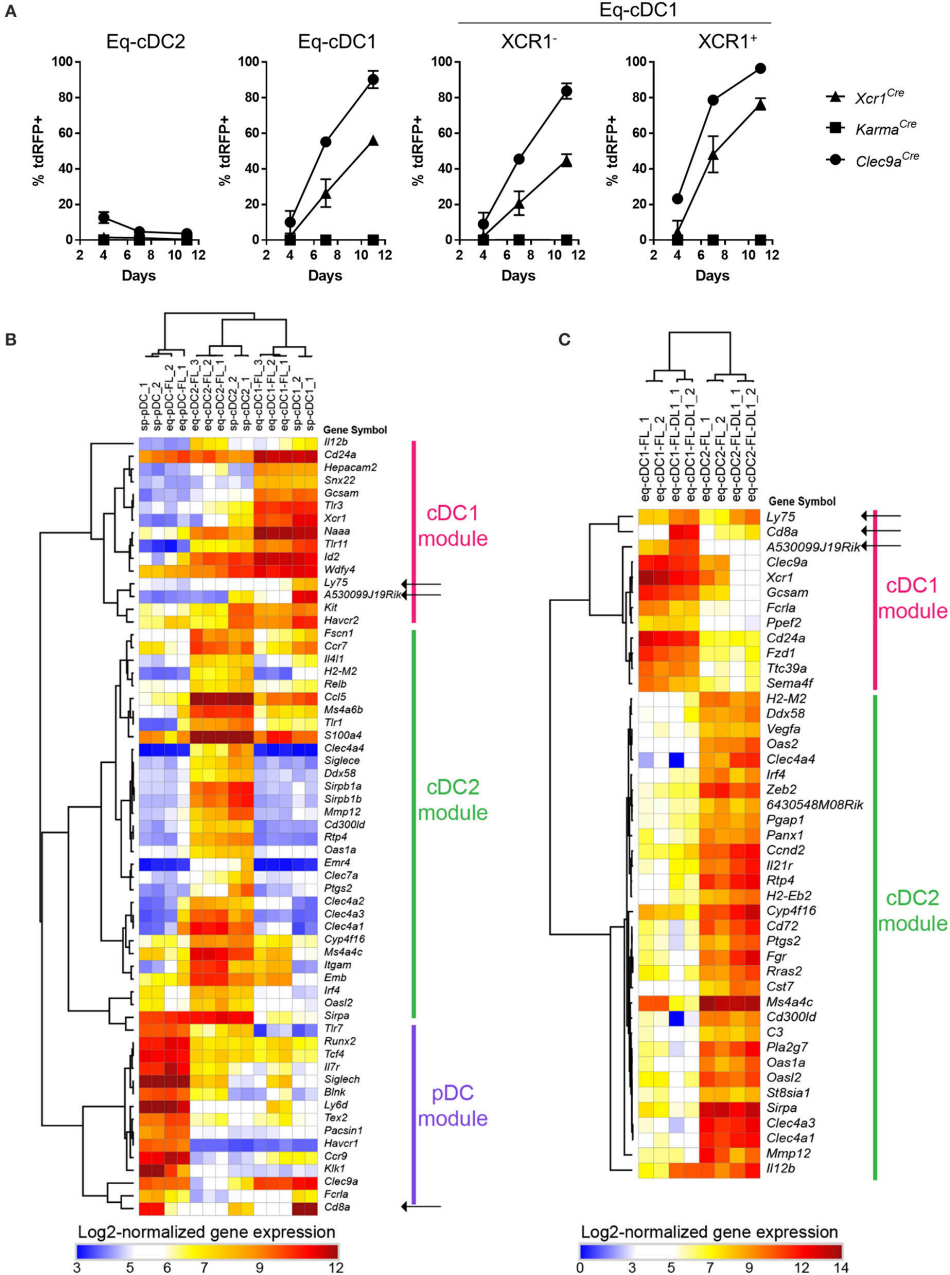
Sequential regulation of *Clec9a, Xcr1* and *Karma* expression during cDC1 differentiation. **(A)** Kinetic analysis of the tdRFP expression in DCs differentiated *in vitro* from *Xcr1*^*Cre*/*wt*^*; Rosa26*^*tdRFP*^^/wt^, *Karma*^*Cre*/*wt*^*; Rosa26*^*tdRFP*^^/wt^ and *Clec9a*^*Cre*/*wt*^*; Rosa26*^*tdRFP*^^/wt^ BM cells cultured with FLT3-L. Eq-cDC2 were gated as CD11c^+^MHC-II^+^CD24^−^SIRPα^+^ cells, and eq-cDC1 as CD11c+ MHC-II^+^CD24^+^SIRPα^−^ cells (Supplementay Figure S4). Data are shown for one experiment representative of two, with three mice per group. **(B,C)** Heatmaps display the expression profiles of archetypical genes previously shown to be selectively expressed in cDC1, cDC2, or pDC. **(B)** Gene expression across DC types either isolated from murine spleens (sp-pDC, sp-cDC1, and sp-cDC2), or derived *in vitro* in standard FLT3-L BM cultures (eq-pDC-FL, eq-cDC1-FL, and eq-cDC2-FL), as assessed with microarrays. **(C)** Gene expression patterns across cDC types derived *in vitro* from FLT3-L BM cultures under standard conditions (-FL) or on DL1-expression OP9 feeder cells (-FL-DL1), as assessed from public RNA-seq data (GEO accession number GSE110577).

We further investigated to which extent our fate mapping mutant mouse models could help refining the differentiation trajectory of cDC1, using as a simple model bone marrow (BM) cells cultured with Fms-like tyrosine kinase 3 ligand (FLT3-L) (38). This model allows *in vitro* generation of three subsets of DCs, which are phenotypically and functionally equivalent to *in vivo* cDC1 (eq-cDC1), cDC2 (eq-cDC2) and pDCs (eq-pDCs) (38, 48–50). To refine the differentiation trajectory of cDC1, we followed the acquisition of the tdRFP signal over time in FLT3-L-differentiated DCs generated from BM of our fate mapping mutant mice (Supplementary Figure S4, Figure 5A). *Xcr1*^*Cre*^ and *Clec9a*^*Cre*^ models allowed efficient recombination in eq-cDC1 (56 vs. 90%) (Figure 5A). Interestingly, *Karma*^*Cre*^ did not present any recombinase activity in any of the DC populations (Figure 5A). Consistently, whereas gene expression profiling of the eq-DC subsets generated in standard BM FLT3-L cultures confirmed their close homology to their *in vivo* counterparts isolated from the spleen (Figure 5B), it also showed that eq-cDC1 lacked expression of the *Karma, Cd8a* and *Ly75* (*Cd205*) genes (Figure 5B, black arrows). This was confirmed using our previously published *Karma* reporter mouse model knocked-in for tdTomato in the 3′UTR of the *Karma* gene (33) (Supplementary Figure S5A). However, these eq-cDC1 acquired *Karma* and CD8α expression upon *in vivo* transfer (Supplementary Figures S5B,C). *Karma* was also expressed in eq-cDC1 differentiated from BM cells cultured with FLT3-L on feeder cells expressing the Notch ligand Delta-like 1 (Figure 5C, black arrows), similarly to what has been recently reported for CD8α and CD205 expression (43). Altogether, these results demonstrate a sequential expression of *Clec9a, Xcr1* and *Karma* during cDC1 ontogeny, with *Clec9a* being induced early starting at the common cDC progenitor stage (21), then followed by *Xcr1* which induction might be initiated already at the pre-cDC1 stage. Likewise to CD8α, *Karma* is acquired at a more advanced differentiation stage that is not reached under classical conditions of DC differentiation from BM in FLT3-L *in vitro* cultures but can be promoted by Notch signaling. This work thus significantly extends two recent studies showing that cDC1 derived *in vitro* from mouse or human hematopoietic precursors with a combination of cytokines and growth factors need additional signals to reach a terminal state of differentiation including acquisition of CD8α expression for mouse cDC1 (43, 51).

### The Cre Expression Under *Xcr1* or *Karma* Promoters Remains cDC1-Specific Upon Infection-Induced Inflammation

The expression of many membrane proteins or transcription factors changes upon inflammation (12, 52). An important incentive for generating *Xcr1*^*Cre*^ and *Karma*^*Cre*^ models, was that the expression of the *Xcr1* and *Karma* genes was specific for cDC1 both at steady state and under inflammatory conditions (53). To confirm this observation based on transcriptomic studies, we examined cDC1-targeting specificity of the *Karma*^*Cre*^ and *Xcr1*^*Cre*^ mouse models in an inflammatory context, namely systemic mouse cytomegalovirus (MCMV) infection, using the *Rosa26*^*tdRFP*^ reporter as a read out. We adapted a gating strategy adapted from (52) to identify inflammatory DCs (InflDCs) in spleen and liver (Supplementary Figure S6). Although we could observe the appearance of InflDCs upon MCMV infection, tdRFP expression remained unchanged in infected animals as compared to control mice, being still essentially confined to the cDC1 population, both in *Xcr1*^*Cre*/*wt*^*; Rosa26*^*tdRFP*^^/wt^ and *Karma*^*Cre*/*wt*^*; Rosa26*^*tdRFP*^^/wt^ mice (Supplementary Figure 6). This demonstrated that both cDC1-targeting models are stable and allow excision of a floxed genomic sequence efficiently and largely selectively in cDC1 at steady state and upon inflammation.

### Germline Recombination of loxP Sequences Is Frequent in the Offspring of *Xcr1^*Cre*^* but Not *Karma^*Cre*^* Mice

Recombination of loxP-flanked genomic sequences in germ cells have been described in many Cre mouse models (54–56). To test whether germline recombination occurs in our cDC1-targeting Cre-driver lines, we backcrossed *Xcr1*^*Cre*/*wt*^*; Rosa26*^*tdRFP*^^/wt^ and *Karma*^*Cre*/*wt*^*; Rosa26*^*tdRFP*^^/wt^ mice to C57BL/6J mice, and analyzed their offspring for ubiquitous tdRFP expression, using blood T and B cells as a readout (Figure 7A). Total or partial germline recombination of the *Rosa26*^*tdRFP*^ locus occurred in 95% of the offspring who had inherited one loxP-flanked allele from *Xcr1*^*Cre*/*wt*^*; Rosa26*^*tdRFP*/*wt*^ male mice (Figure 7B). Germline recombination occurred with the same frequency irrespective of the segregation of the paternal *Cre* and loxP-flanked alleles in the offspring, demonstrating that this process occurred during meiosis rather than in the embryo. No germline recombination was observed when both alleles were from maternal germ cells (Figure 7B). In ongoing crosses using the *Xcr1*^*Cre*^ mouse model with different loxP-flanked mouse strains, we could also observe germline recombination in the progeny even when the floxed alleles were brought together with the *Xcr1*^*Cre*^ allele by the maternal gamete (data not shown). This indicates that, contrary to what the results of the *Rosa26*^*tdRFP*^^/wt^ backcross appears to suggest, off-target activity of the Cre recombinase in germline is not a gender effect. Additionally, the incidence of germline recombination depended on the loxP-flanked allele (data not shown). No occurrence of germline recombination was detected so far for the *Karma*^*Cre*^ model (Figure 7B). Therefore, the *Karma*^*Cre*^ model might be more appropriate than the *Xcr1*^*Cre*^ model to obtain rapidly mice in which cDC1 are inactivated for candidate genes, through conventional breeding strategies. Germline recombination in the offspring should however always be assessed for any novel loxP-flanked allele.

**Figure 6:**
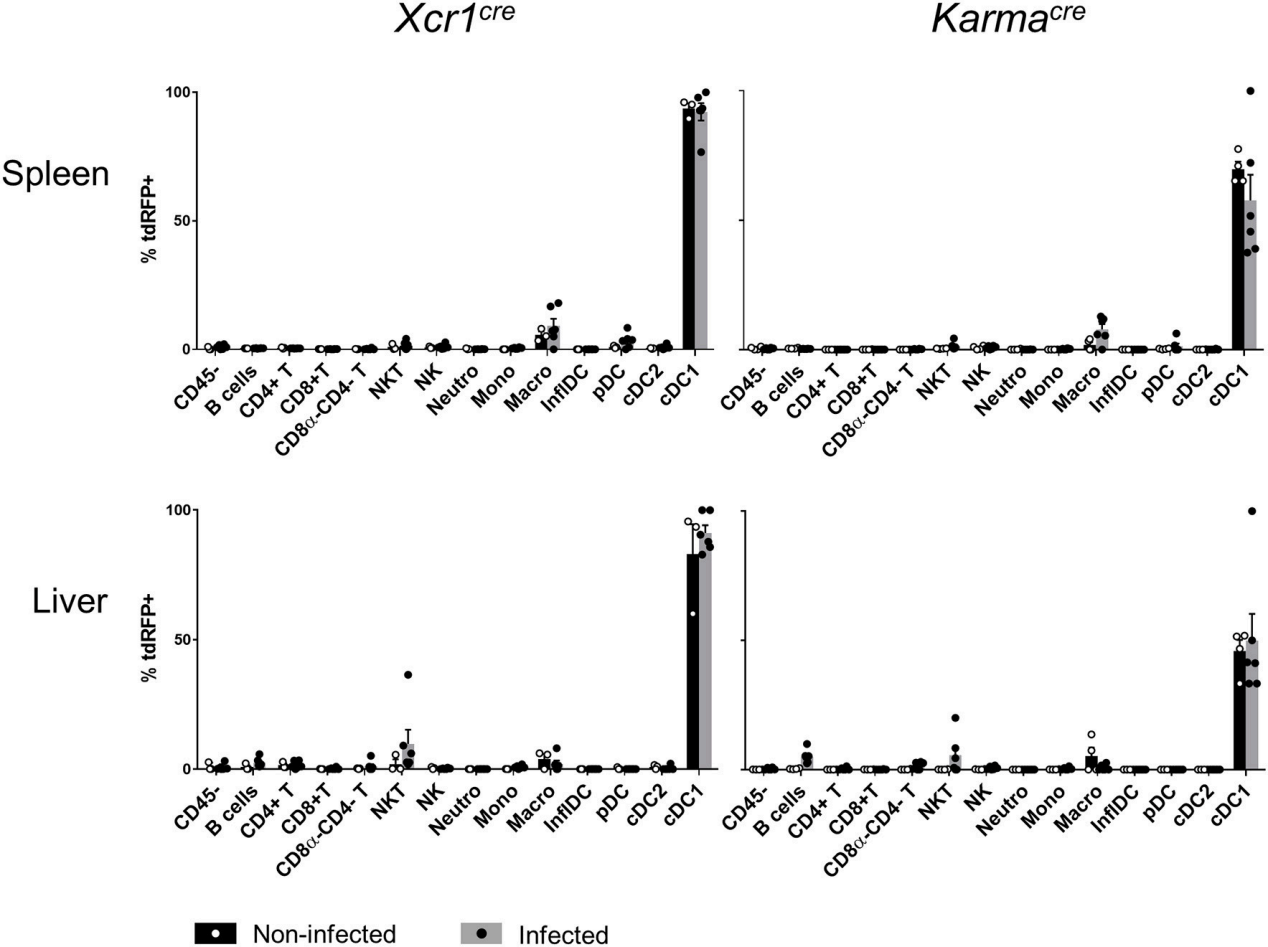
*Xcr1* and *Karma* expressions remain confined to cDC1 upon virus-induced inflammatory responses. Flow cytometry analysis of the tdRFP expression by different immune cell populations as in Figure 2A, from spleen, CLNs, and liver of *Xcr1*^*Cre*/*wt*^*; Rosa26*^*tdRFP*^^/wt^, and *Karma*^*Cre*/*wt*^*; Rosa26*^*tdRFP*^^/wt^ mice 2 days after MCMV infection. Cell population gating strategy detailed in Supplementay Figure S3. Data show one dot per individual value, mean +/- SEM per group, and are from two pooled experiments with at least three individuals per group of infected mice. Statistical analyses were performed using nonparametric Mann-Whitney tests when possible, and the difference between non infected and infected was non-significant in each cell population. NI, non-infected; InflDCs, inflammatory DCs.

**Figure 7:**
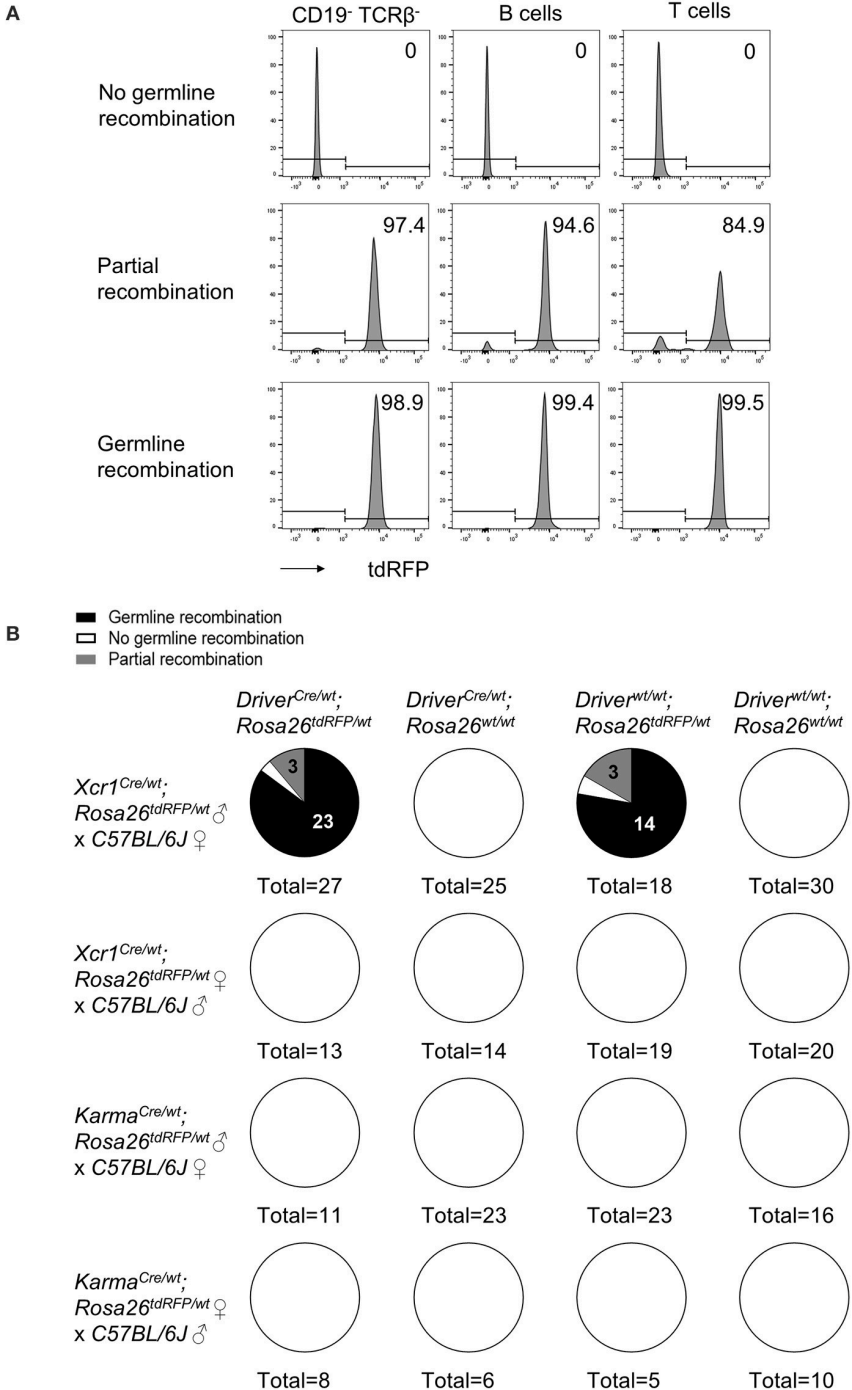
The Cre expression under *Xcr1* but not *Karma* promoter efficiently recombines Lox-sequences in germ cells. **(A)** Representative flow cytometry analysis of tdRFP expression in CD19^−^ TCRβ^−^, B and T cells in blood of mice with no germline recombination, partial recombination, and germline recombination. One sample representative of each is shown. **(B)** Analysis of germline recombination in offspring from backcrosses of *Xcr1*^*Cre*/*wt*^*; Rosa26*^*tdRFP*^^/*wt*^ and *Karma*^*Cre*/*wt*^*; Rosa26*^*tdRFP*^^/wt^ mice of both sexes with wild-type (*WT;* C57BL/6J) mice. Germline recombination shown here occurred when both *Cre* and *floxed* alleles were of paternal origin. However, with other type of flox constructs, we regularly observed occurrences of germline recombination when both alleles were brought together, either by the father or by the mother.

## Discussion

*Xcr1* and *A530099j19rik* (*Karma/Gpr141b)* genes code for the chemokine receptor XCR1 and for the putative G protein-coupled receptor Gpr141b, respectively, and are among the core gene signature specifically identifying mouse cDC1 throughout the organism (10, 22–25, 33). We have inserted an IRES-Cre cassette into the 3′ UTR of the *Xcr1* and *Karma* coding exon to generate *Xcr1*^*Cre*^ and *Karma*^*Cre*^ mouse models, respectively. In this study, we have characterized the efficiency and specificity of Cre-mediated recombination in these novel mouse models at steady state and upon viral infection, comparing them to the *Clec9a*^*Cre*^ model. To the best of our knowledge, this study demonstrated that our novel *Xcr1*^*Cre*^ and *Karma*^*Cre*^ mouse models are the most trustful and robust for the genetic tracking and manipulation of cDC1 *in vivo*.

Amongst the three Cre-driver mouse models examined, *Xcr1*^*Cre*^ model is the most efficient and specific for fate mapping all cDC1 regardless of the tissues examined. The *Karma*^*Cre*^ model is rather specific for cDC1 when compared with *Clec9a*^*Cre*^ mouse, but much less efficient than the *Xcr1*^*Cre*^ model. Unexpectedly, a fraction of CD4^+^ T cells is labeled with tdRFP in the *Xcr1*^*Cre*^;*Rosa26*^*tdRFP*^^/wt^ mouse (Figure 2) without expressing any detectable XCR1 at their cell surface (Supplementary Figure S1E). Further analysis need to be conducted to determine whether XCR1 was transiently turned on in the distant progenitors of these cells or on the contrary during their terminal differentiation. Interestingly, the proportion of *Xcr1*^*cre*^ fate-mapped CD4^+^ T cells was much higher in the skin than in the other organs examined, suggesting that these cells may be polarized toward specific functions and/or develop under instructive signals encountered preferentially in barrier organs. Further studies will be needed to test these hypotheses. In the skin of the *Karma*^*Cre*^*; Rosa26*^*tdRFP*^^/wt^ mouse model, the vast majority of the tdRFP^+^ cells were of mast cell origin (Figure 3B). Microarray data released recently by the Immgen consortium (https://www.immgen.org) show that mast cells from the skin, peritoneal cavity, trachea and esophagus express high level of the *Karma* gene (Supplementary Figure S3), confirming our observation. To the best of our knowledge, this is the first report of a gene that is selectively shared by both cDC1 and mast cells. The *Karma*^*Cre*^ mice will therefore be of special interest to researchers aiming at genetically manipulating mast cells in tissues. In all tissues and in all mouse models examined, no tdRFP expression was detected in the CD45-negative cells present in cell suspensions (Figure 2). Although, we did not examine tdRFP expression in other non-hematopoietic cells, it is unlikely that some of these cell types would be targeted in *Xcr1*^*Cre*^ or *Karma*^*Cre*^ mice considering that the *Xcr1* and *Gp141b* genes were not expressed outside of the hematopoietic system in all of the transcriptomic databases we queried.

To inactivate specifically a candidate gene in cDC1, both alleles of this candidate gene should be excised. This requires a breeding strategy in which one *Cre* allele and one floxed allele of the gene to be inactivated are brought by the same germ cells, where unexpected recombination could occur. We have tested the frequency of germline recombination for both the *Xcr1*^*Cre*^ and *Karma*^*Cre*^ models. Only the *Xcr1*^*Cre*^ model showed adventitious Cre activity in germ cells resulting in progeny with recombined *Rosa26*^*tdRFP*^ locus in all their cells. Interestingly, this was paternal inherited in this specific experimental setting. However, this may depend on the loxP-flanked construct, as we had events of germline recombination transmitted by females for other floxed genes than the *Rosa26*^*tdRFP*^ reporter. Therefore, to reach specific recombination in cDC1 using the *Xcr1*^*Cre*^ model, the *Cre* allele and the loxP-flanked allele should be inherited from different parents. We recommend breeding one parent homozygous for both the *Cre* allele and a null allele of the target gene, to another parent homozygous for the floxed allele of the target gene. Each investigator using the *Xcr1*^*Cre*^ model should always test their progeny for unexpected off-target recombination. A recent publication strongly suggested to include, in each experimental procedure of publication using Cre mouse models, detailed procedures about the breeding strategies used and the method the investigators applied to detect any unexpected and unspecific recombination (56).

Our genetic tracing of cDC1 *in vivo* in the spleen (Figure 4), or *in vitro* in FLT3-L-differentiated BM-DC cultures (Figure 5A) revealed heterogeneity in the cDC1 population. The differential expression of *Xcr1* and *Karma* genes within the cDC1 population qualifies *Xcr1*^*Cre*^ and *Karma*^*Cre*^ mouse models as powerful tools to describe further these cDC1 subsets *in vivo*. *In vitro*, BM-DCs derived from *Karma*^*Cre*^ mice did not show any sign of Cre activity (Figure 5A), confirming that the *Karma* gene is not transcriptionally active in these cells as directly assessed through their gene expression profiling (Figure 5B), akin to *Cd8a* or *Ly75* (*Dec205*) (43). We show here that expression of the *Karma* gene on cDC1 requires accessory signals, which can be provided upon *in vivo* transfer, or *in vitro* by Notch signaling likewise to what has been recently reported for *Cd8a* and *Ly75* (43). Of note, in FLT3-L *in vitro* BM-DC culture, detection in eq-cDC1 of the activity of the Cre recombinase as readout by tdRFP expression seemed to be delayed over time as compared to cell surface acquisition of XCR1 (Figure 5), although the *Cre* and *Xcr1* genes were expressed under the same promoter from one bi-cistronic mRNA. This might be explained by a delayed translation of the *Cre* gene as compared to *Xcr1*, or because efficient recombination of DNA by the Cre requires time. This latter case might especially apply to the *Rosa26*^*tdRFP*^ reporter mouse line used in this study, because it was engineered as requiring two consecutive rounds of Cre-excision to generate detectable tdRFP signal, in order to limit any leaky transcription of the fluorescent reporter gene across the stopper at steady state (35). This contrasts to most reporter lines which require only one sequence of recombination to emit signal (57, 58) and must therefore require lower and/or less sustained Cre activity to allow recombination. Breeding the *Xcr1*^*Cre*^ model with a Cre-reporter mouse which is easily recombined (57) might allow a better synchronization of XCR1 surface expression with Cre activity.

Our results advanced our understanding of the differentiation trajectory of cDC1, and validated the *Xcr1*^*Cre*^ mouse model as a robust tool to inactivate genes selectively in cDC1 either *in vivo* or *in vitro* using BM-derived DC cultures. Future use of these mutant mouse models will undoubtedly boost the advancing of our understanding of the biology of cDC1.

## Author Contributions

MD and KC: Conceptualization. RM, CW, BM, MD, and KC: Methodology, Validation, Formal Analysis. RM, CW, SG, MA, YA, CS, and AF: Investigation. T-PV, MD: bioinformatics analyses. BM: Mouse model construction supervision. RM, KC: Visualization. RM, KC, and MD: Writing. All authors: Editing. BM, MD, and KC: Project Supervision and Administration.

### Conflict of Interest Statement

The authors declare that the research was conducted in the absence of any commercial or financial relationships that could be construed as a potential conflict of interest.
