# Supplementary material for: Novel Cre-Expressing Mouse Strains Permitting to Selectively Track and Edit Type 1 Conventional Dendritic Cells Facilitate Disentangling Their Complexity in vivo
Source: Front Immunol. 2018 Dec 4;9:2805. doi: 10.3389/fimmu.2018.02805 (PMC6288293; doi:10.3389/fimmu.2018.02805)
Supplement: Supplementary file 1 [file Data_Sheet_1.docx]

Supplementary Material

Novel Cre-expressing mouse strains permitting to selectively track and edit type 1 conventional dendritic cells facilitate disentangling their complexity *in vivo*

Raphaël MATTIUZ^1^, Christian WOHN^1^, Sonia GHILAS^1^, Marc AMBROSINI^1^, Yannick O. ALEXANDRE^1^, Cindy SANCHEZ^1^, Anissa FRIES^1^, Thien-Phong VU MANH^1^, Bernard MALISSEN^1,2^, Marc DALOD^1*^, Karine CROZAT^1*^

^1^ Aix Marseille Univ, CNRS, INSERM, Centre d’Immunologie de Marseille-Luminy, Turing Center for Living Systems, Marseille, France.

^2^ Aix Marseille Univ, CNRS, INSERM, Centre d’Immunophénomique, Marseille, France

**Correspondence:**Karine CROZAT, [crozat@ciml.univ-mrs.fr](mailto:crozat@ciml.univ-mrs.fr) and Marc DALOD [dalod@ciml.univ-mrs.fr](mailto:dalod@ciml.univ-mrs.fr)

# Supplementary Tables

**Supplementary Table 1: List of the antibodies, their clones and conjugates used in this study.**

| **Antibody** | **Clone** | **Conjugates** | **Company** | **Dilution** |
| --- | --- | --- | --- | --- |
| **CD3ε** | M5/114.15.2 | Alexa700 | BioLegend | 1/200 |
| **CD3ε** | 145-2C11 | BV510 | BD Biosciences | 1/200 |
| **CD4** | RM4-5 | PerCP-Cy5.5 | BD Biosciences | 1/200 |
| **CD8α** | 53-6.7 | PE-Cy7 | BD Biosciences | 1/400 |
| **CD8α** | 53-6.7 | V450 | BD Biosciences | 1/200 |
| **CD8α** | 53-6.7 | FITC | BD Biosciences | 1/200 |
| **CD11b** | M1/70 | BUV395 | BD Biosciences | 1/400 |
| **CD11c** | HL3 | BUV737 | BD Biosciences | 1/200 |
| **CD11c** | N418 | BV785 | BioLegend | 1/200 |
| **CD19** | 1D3 | BV510 | BD Biosciences | 1/200 |
| **CD24** | M1/69 | eFluor450 | eBioscience | 1/1000 |
| **CD24** | M1/69 | Biotin | BioLegend | 1/400 |
| **CD24** | M1/69 | PE-Cy7 | BD Biosciences | 1/1000 |
| **CD26** | H194-112 | BV786 | BD Biosciences | 1/100 |
| **CD45.2** | 104 | BV605 | BioLegend | 1/200 |
| **CD45.2** | 104 | BUV737 | BD Biosciences | 1/400 |
| **CD49b** | DX5 | FITC | BD Biosciences | 1/200 |
| **CD64** | X54-5/7.1 | BV711 | BioLegend | 1/200 |
| **CD64** | X54-5/7.1 | BV421 | BioLegend | 1/200 |
| **CD64** | X54-5/7.1 | Alexa647 | BD Biosciences | 1/500 |
| **CD86** | GL1 | APC | BD Biosciences | 1/400 |
| **CD103** | M290 | PerCP-Cy5.5 | BD Biosciences | 1/400 |
| **CD115** | AFS98 | BV711 | BioLegend | 1/400 |
| **CD117** | 2B8 | PE-Cy7 | BD Biosciences | 1/200 |
| **CD172a** | P84 | FITC | BD Biosciences | 1/200 |
| **CD172a** | P84 | APC-Cy7 | BioLegend | 1/100 |
| **ESAM** | 1G8/ESAM | APC | BioLegend | 1/200 |
| **FcεRIα** | MAR-1 | PacificBlue | BioLegend | 1/200 |
| **F4/80** | BM8 | APC | BioLegend | 1/400 |
| **F4/80** | BM8 | BV605 | BioLegend | 1/200 |
| **F4/80** | BM8 | PE-Cy7 | BioLegend | 1/400 |
| **Ly-6C** | AL-21 | APC-Cy7 | BD Biosciences | 1/1000 |
| **Ly-6C** | AL-21 | FITC | BD Biosciences | 1/600 |
| **Ly-6G** | 1A8 | APC-Cy7 | BioLegend | 1/400 |
| **Ly-6G** | 1A8 | BV510 | BD Biosciences | 1/200 |
| **MerTK** | REA477 | Biotin | Miltenyi Biotec | 1/10 |
| **MHC-II** | M5/114.15.2 | Alexa700 | BioLegend | 1/400 |
| **NK1.1** | PK136 | PE-Cy7 | BD Biosciences | 1/200 |
| **NKp46** | 29A1.4 | BV510 | BD Biosciences | 1/200 |
| **Siglec-H** | 551 | PerCP-Cy5.5 | BioLegend | 1/200 |
| **Siglec-H** | 551 | PacificBlue | BioLegend | 1/200 |
| **ST2** | RMST2-33 | Biotin | eBioscience | 1/200 |
| **XCR1** | ZET | BV650 | BD Biosciences | 1/1000 |

## Supplementary Figures

**
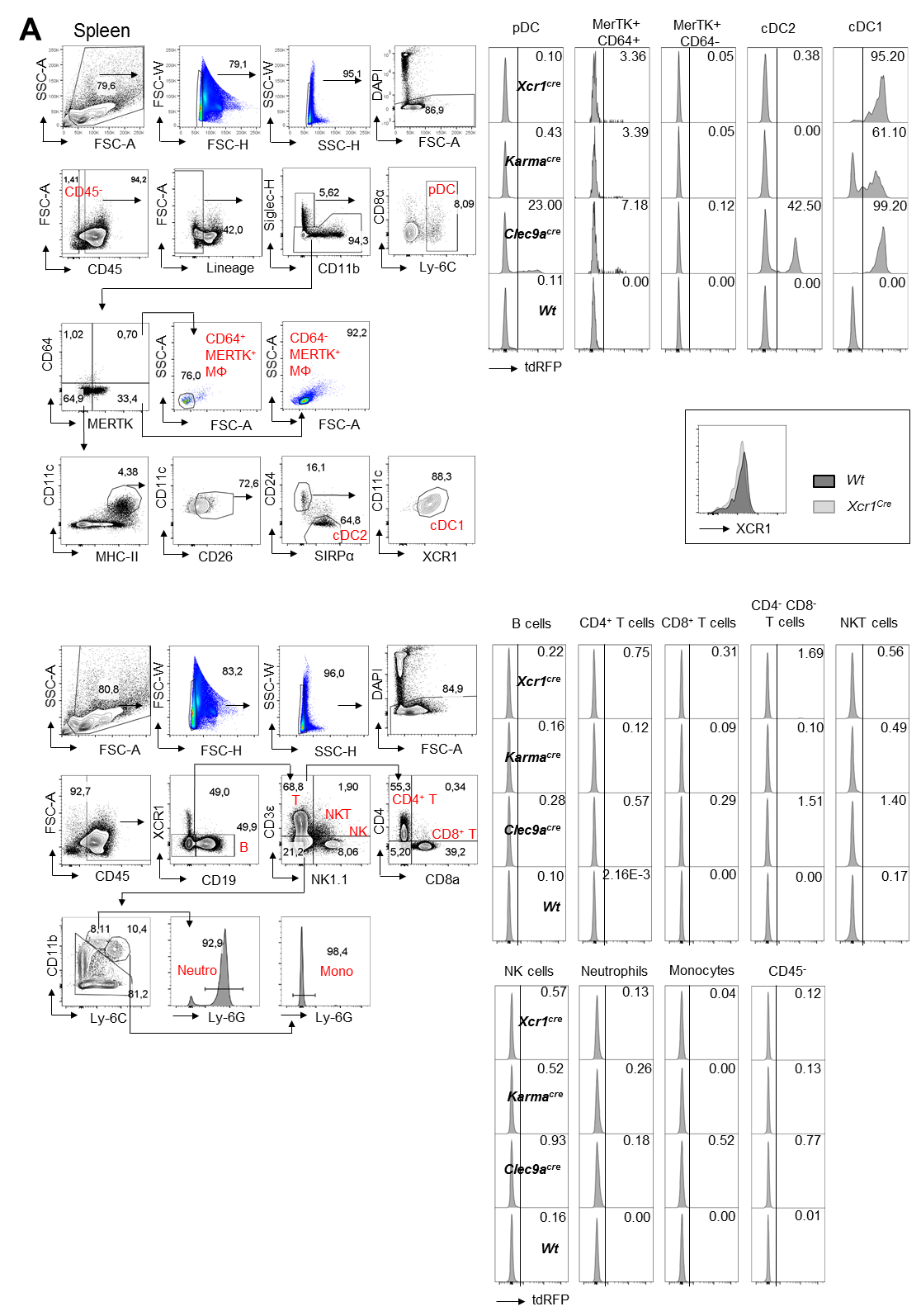
**

**
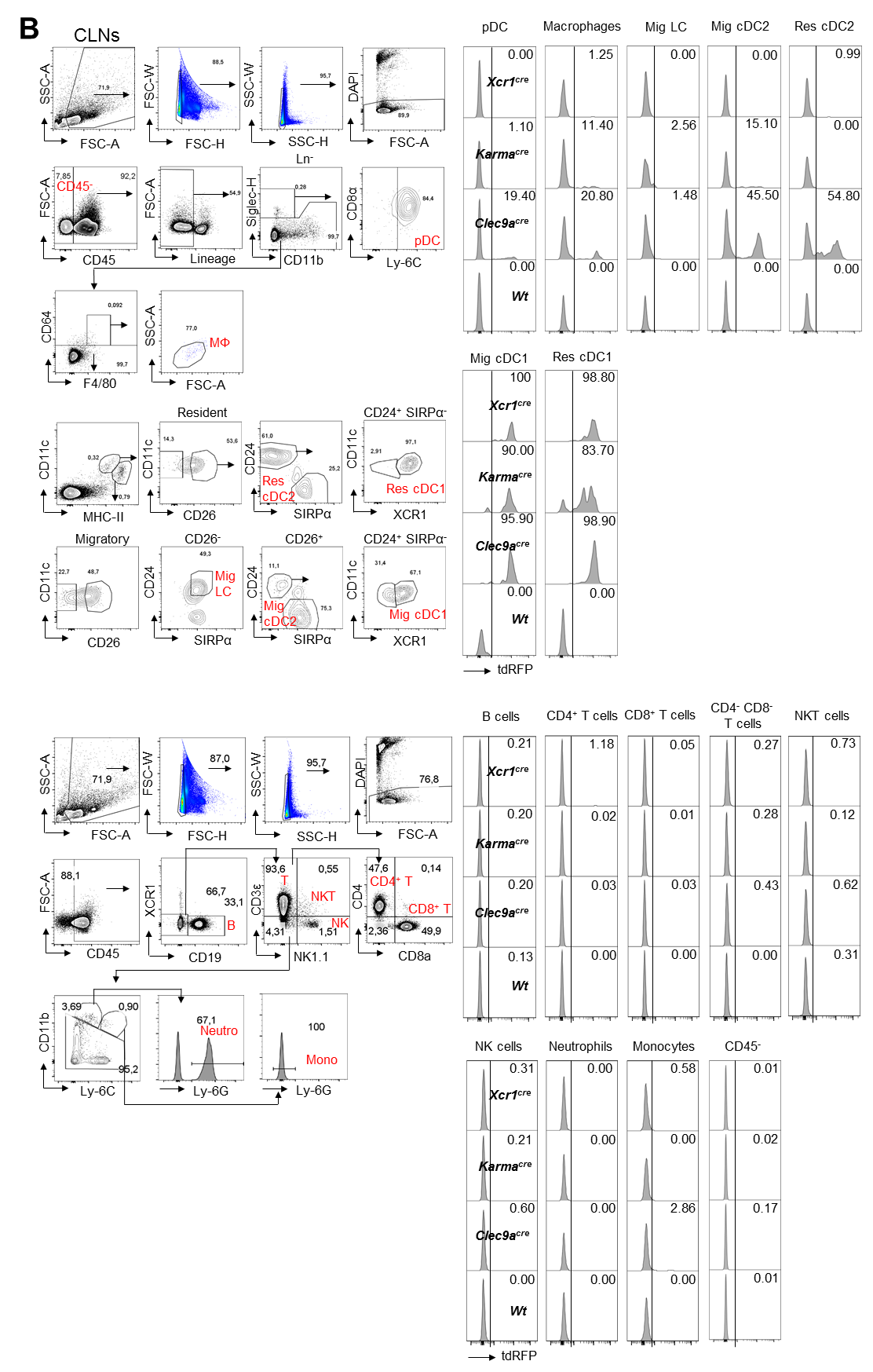
**

**
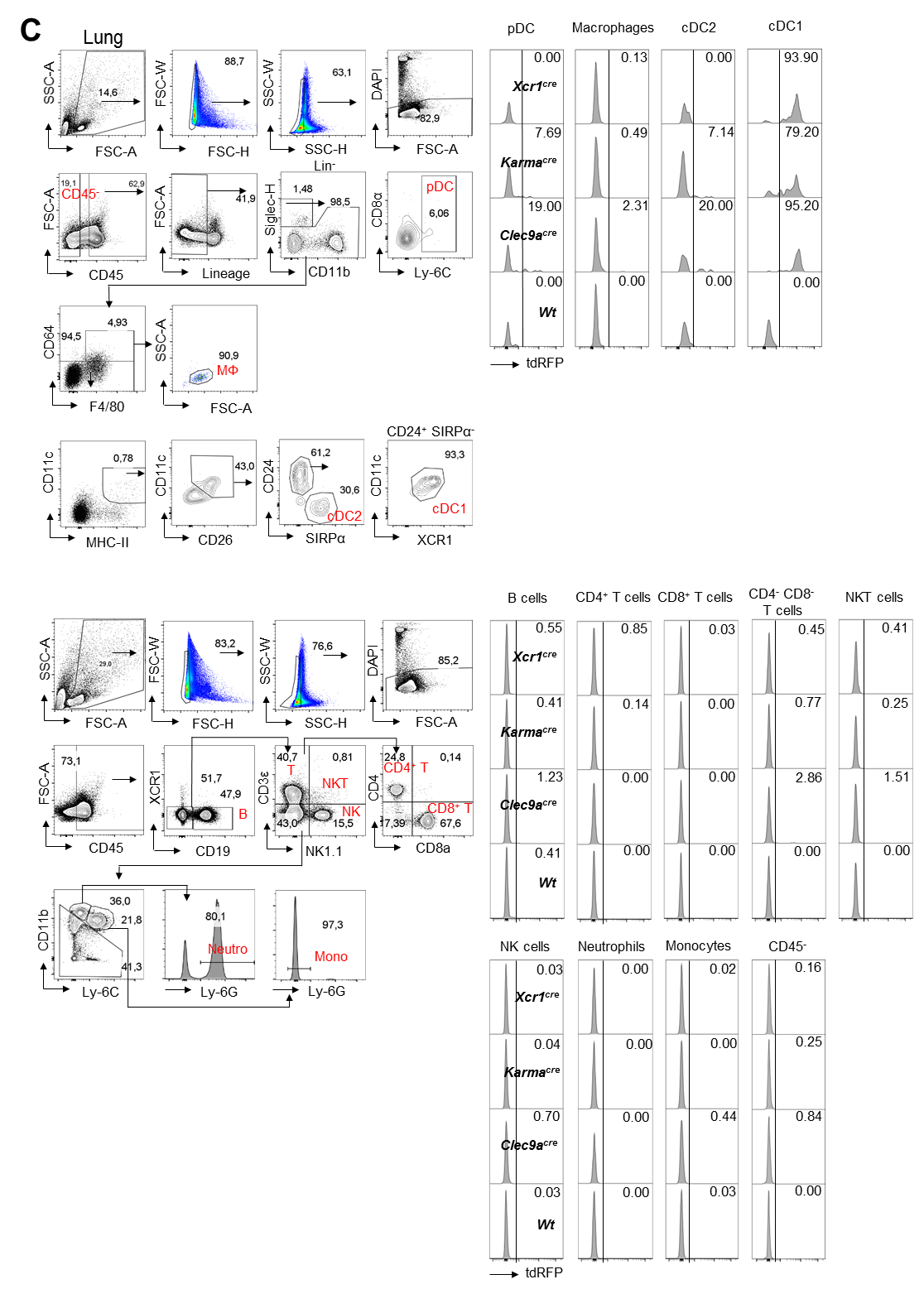
**

**
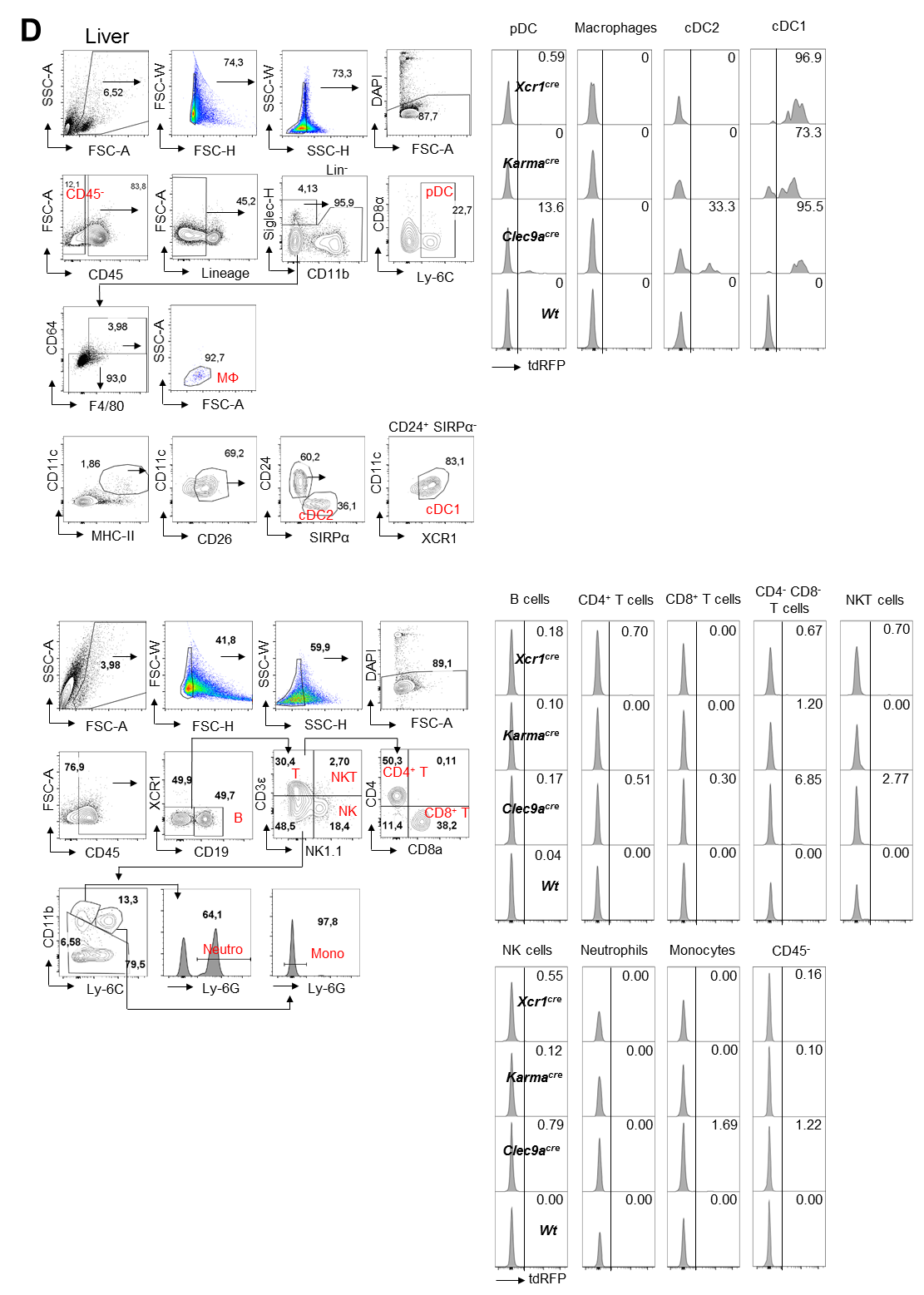
**

**
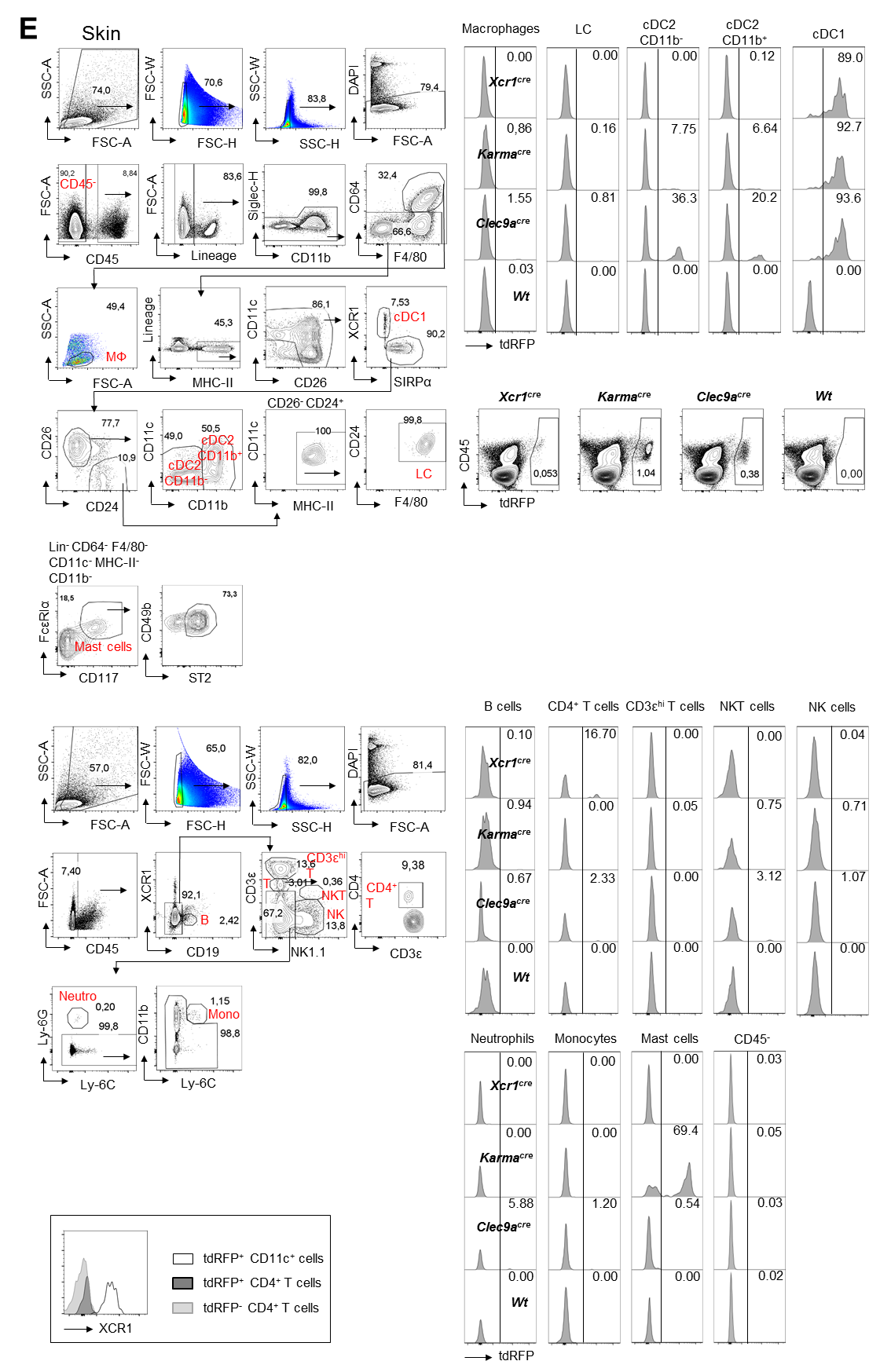
**

**Supplementary Figure S1 (related to Figures 2 and 3): Gating strategies used to identify lymphoid and myeloid cell populations in spleen, CLNs, lungs, liver and skin of *Xcr1^Cre/wt^;Rosa26^tdRFP^*^/^*^wt^*, *Karma^Cre/wt^;Rosa26^tdRFP^*^/^*^wt^* and *Clec9a^Cre/wt^;Rosa26^tdRFP^*^/^*^wt^* mice, and level of RFP expression in these populations.** Left, gating strategies used to define each cell population analyzed in Fig.2. One sample representative of all is shown. Right, RFP expression in these populations isolated from *Xcr1^Cre/wt^;Rosa26^tdRFP^*^/^*^wt^*, *Karma^Cre/wt^;Rosa26^tdRFP^*^/^*^wt^*, *Clec9a^Cre/wt^;Rosa26^tdRFP^*^/^*^wt^* and *Wt* mice. One mouse representative of three is shown for each genotype. A) Spleen; B) CLNs, C) Lungs, D) Liver and E) Skin. Data are shown for one experiment representative of three. This figure also includes: the level of expression of XCR1 in *Wt* vs *Xcr1^Cre^* cDC1 confirming that the genetic manipulation does not significantly alter the endogenous expression of XCR1 (A, top); the proportion of tdRFP+ cells in the skin of *Xcr1^Cre/wt^;Rosa26^tdRFP^*^/^*^wt^*, *Karma^Cre/wt^;Rosa26^tdRFP^*^/^*^wt^*, *Clec9a^Cre/wt^;Rosa26^tdRFP^*^/^*^wt^* and *Wt* mice (E, top); and XCR1 expression by tdRFP^+^ and tdRFP^-^ CD4^+^ T cells in the skin of *Xcr1^Cre/wt^;Rosa26^tdRFP^*^/^*^wt^* mice (E, bottom).


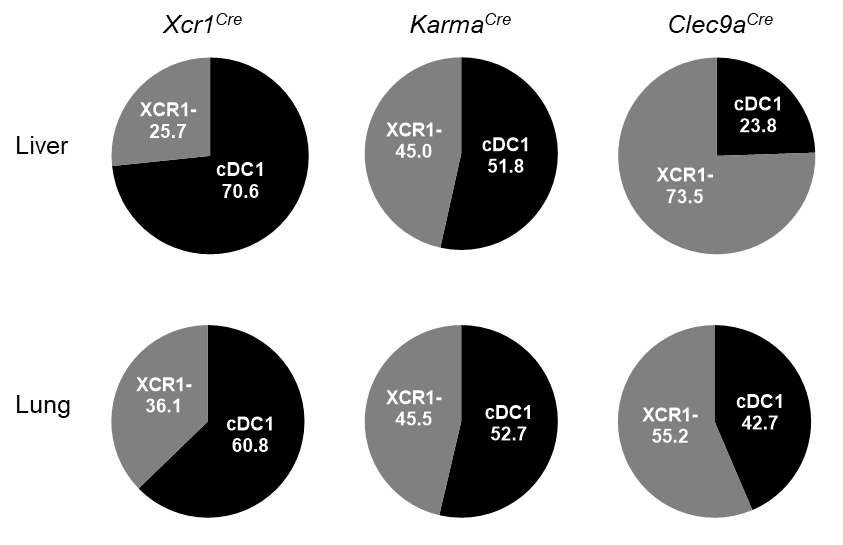


**Supplementary Figure S2 (related to Figure 3): Proportion of cDC1 within tdRFP^+^ cells in the liver and the lungs of *Xcr1^Cre/wt^;Rosa26^tdRFP^*^/^*^wt^*, *Karma^Cre/wt^;Rosa26^tdRFP^*^/^*^wt^* and *Clec9a^Cre/wt^;Rosa26^tdRFP^*^/^*^wt^* mice.** The mean of three mice per group is shown. Data are shown for one experiment representative of three with three mice per group.

**
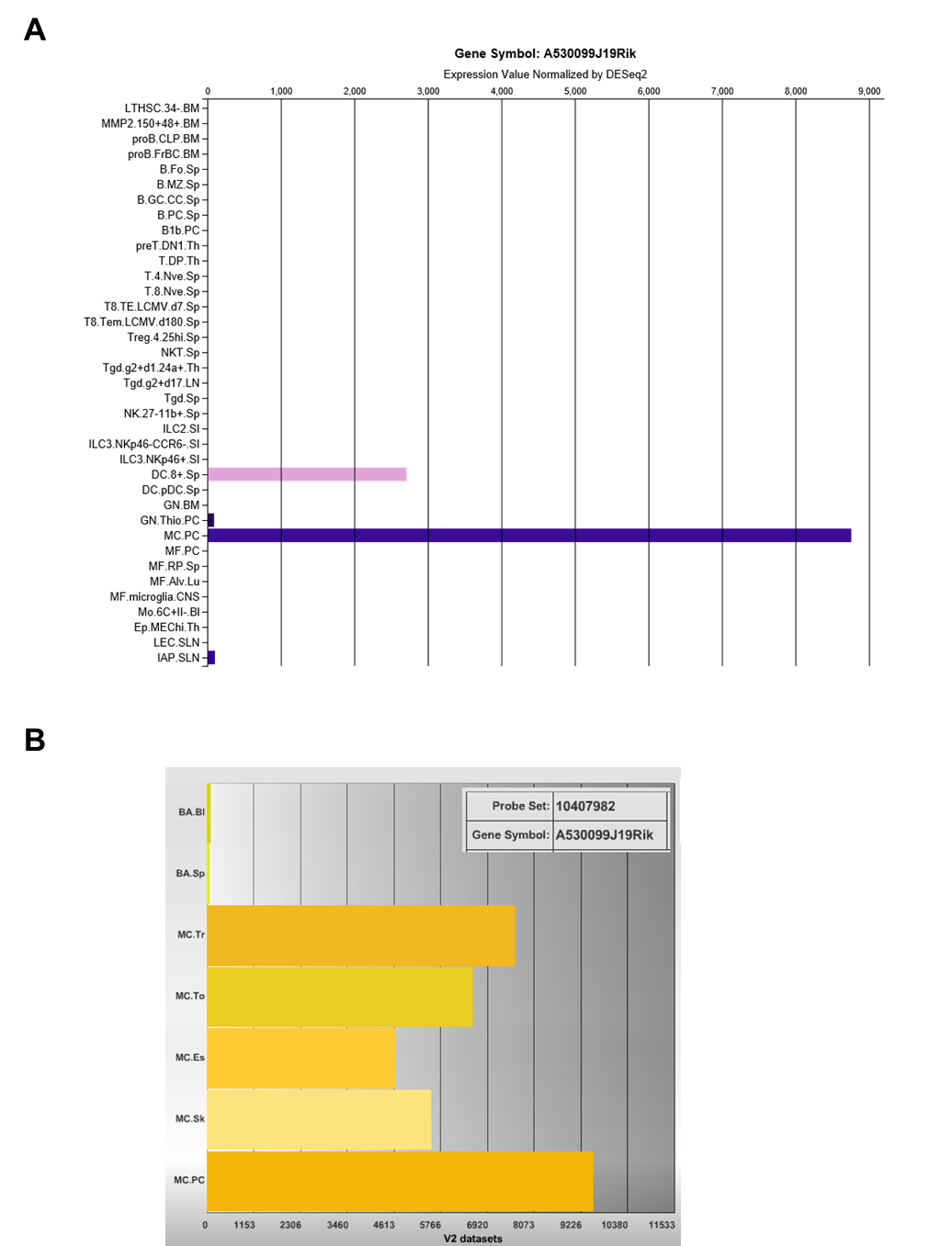
Supplementary Figure S3 (related to Figure 3): Besides cDC1, *A530099j19rik*/*Karma*/*Gpr141b* is specifically expressed by mast cells.** (A) Screenshot of the RNAseq analysis of *A530099j19rik*/*Karma*/*Gpr141b* expression in different key immune cell populations, as generated by the Immgen Consortium. DC.8+.Sp, CD8a^+^ cDC1 isolated from the spleen; MC.PC, mast cells isolated from the peritoneal cavity. (B) Screenshot of the microarray analysis of *A530099j19rik*/*Karma*/*Gpr141b* expression in basophils (BA) isolated from the blood (Bl) and the spleen (Sp), and in mast cells isolated from the trachea (Tr), tongue (To), esophagus (Es), skin (Sk) and peritoneal cavity (PC), as generated by the Immgen Consortium.


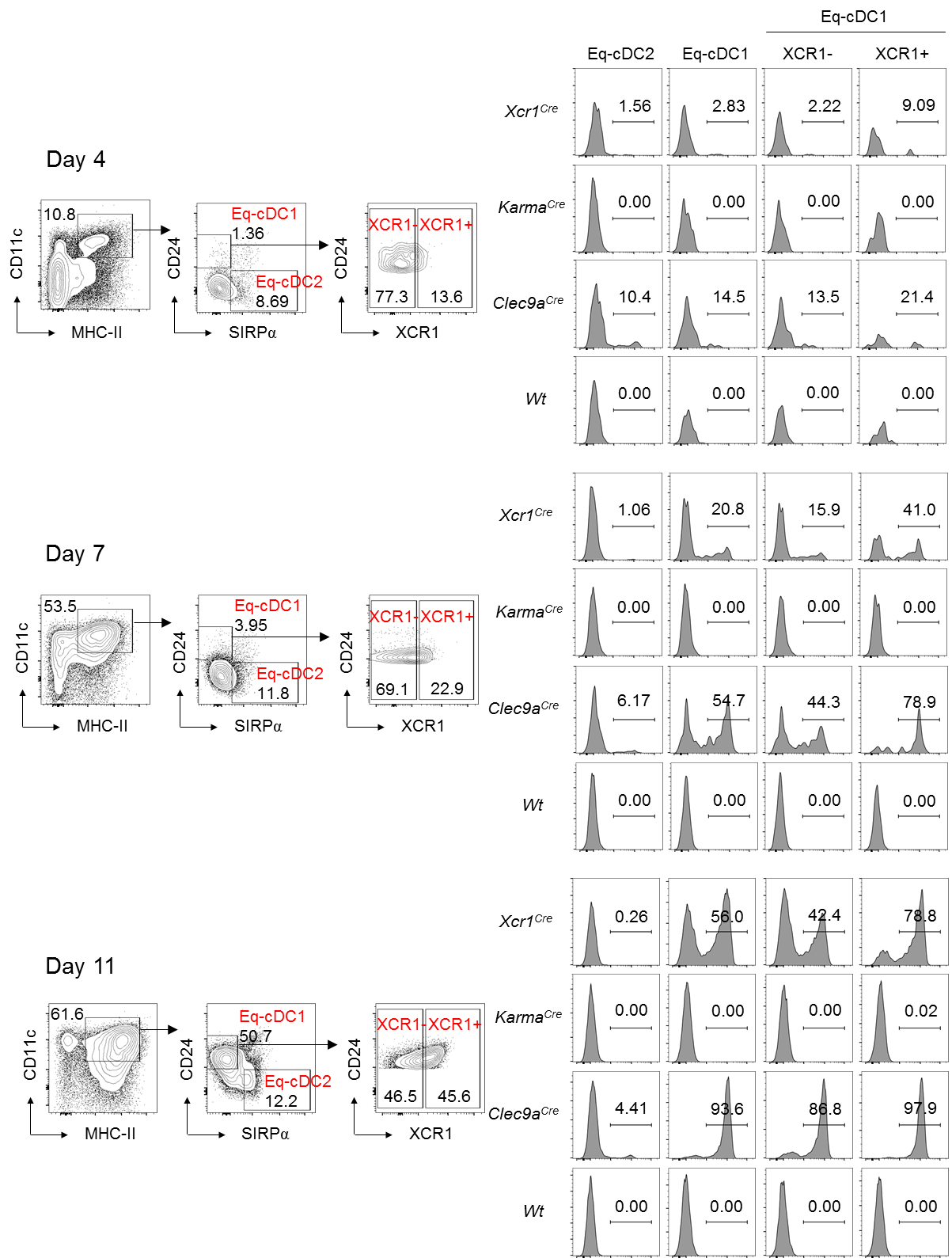


**Supplementary Figure S4 (related to Figure 5): Gating strategies used to identify FLT3-L BM-differentiated eq-cDC1 and eq-cDC2 from *Xcr1^Cre/wt^;Rosa26^tdRFP^*^/^*^wt^*, *Karma^Cre/wt^;Rosa26^tdRFP^*^/^*^wt^* and *Clec9a^Cre/wt^;Rosa26^tdRFP^*^/^*^wt^* mice, and level of tdRFP expression in these populations.** Left, gating strategies used to define each cell population analyzed in Fig.4. One sample representative of all is shown for each day analyzed. Right, tdRFP expression in these populations differentiated from *Xcr1^Cre/wt^;Rosa26^tdRFP^*^/^*^wt^*, *Karma^Cre/wt^;Rosa26^tdRFP^*^/^*^wt^*, *Clec9a^Cre/wt^;Rosa26^tdRFP^*^/^*^wt^* and *Wt* mice. One mouse representative of three is shown for each genotype. Data are shown for one experiment representative of two.

**
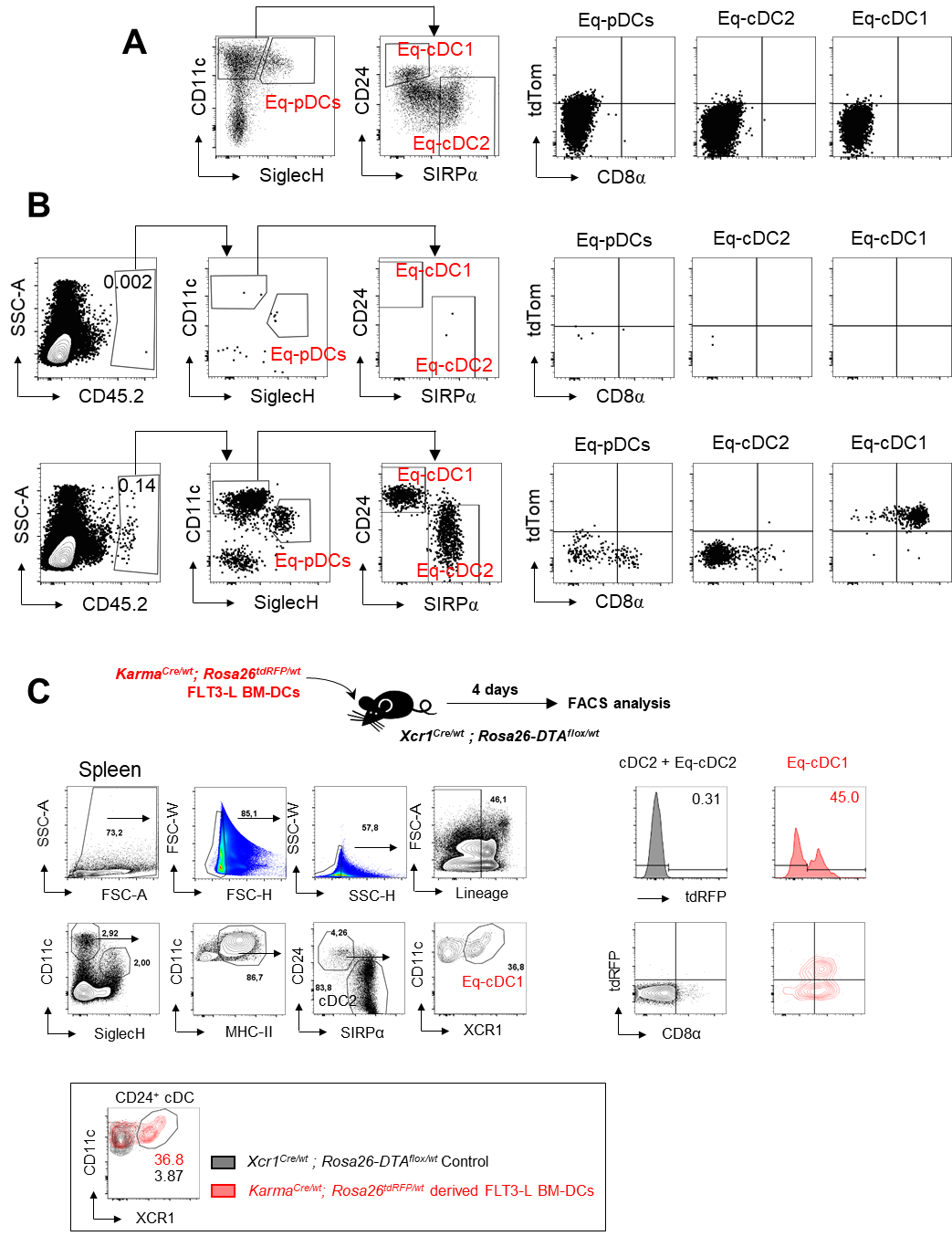
**

**Supplementary Figure S5 (related to Figure 5): Expression of CD8α and *Karma* by eq-cDC1 generated from FLT3-L-differentiated BM-DCs is acquired upon *in vivo* transfer.** (A) CD8α and *Karma* expression by equivalent pDC, cDC1 and cDC2 populations generated *in vitro* after 5 days differentiation of *Karma-tdTomato-hDTR* BM with FLT3-L. (B) CD8α and *Karma* expression by *Karma-tdTomato-hDTR* equivalent pDCs, cDC1 and cDC2, after *in vivo* transfer. 2 x 10^6^ FLT3-L-differentiated bulk BM-DCs generated in (A) were transferred (bottom) or not (top) into a CD45.1 recipient mouse. CD45.2 cells were analyzed from spleens of recipients 5 days after. *Karma* expression was determined by the expression of the tdTomato reporter. Data are shown for one sample representative of three for the *in vivo* transfer. (C) Acquisition of CD8α and tdRFP expression by equivalent cDC1 derived from *Karma^Cre/wt^;Rosa26^tdRFP/wt^* BM with FLT3-L, after *in vivo* transfer. 7 days after incubation with FLT3-L, 2 x 10^6^ bulk BM-DCs were transferred into *Xcr1^Cre/wt^;Rosa26-DTA^flox/wt^* recipient mice, which are selectively depleted of all cDC1. cDCs were analyzed from spleens of recipients 4 days after. *Karma* expression was determined by the expression of the tdRFP. Data are shown for one sample representative of four.


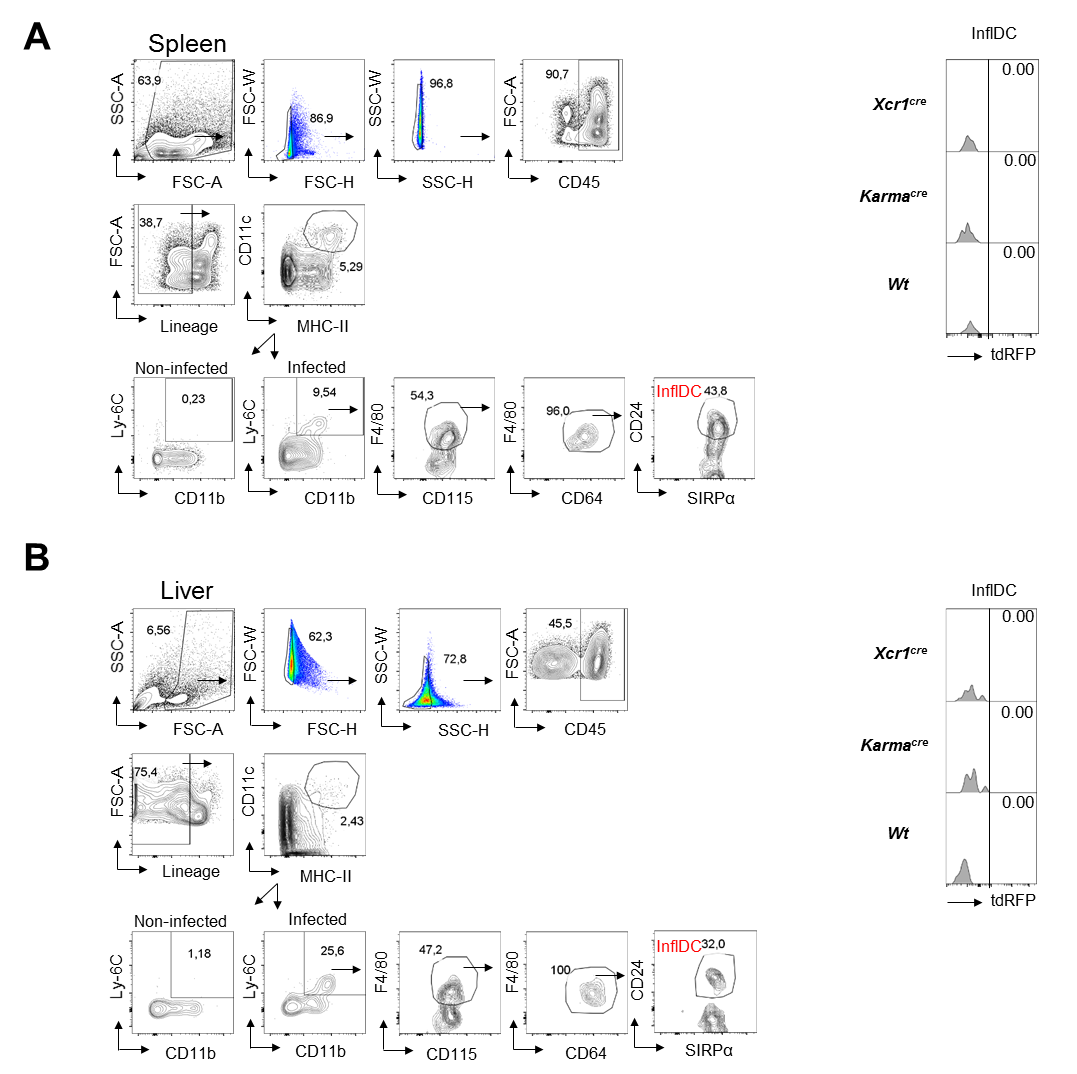


**Supplementary Figure S6 (related to Figure 6): Gating strategies used to identify inflammatory-induced DCs in spleen and liver of *Xcr1^Cre/wt^;Rosa26^tdRFP^*^/^*^wt^* and *Karma^Cre/wt^;Rosa26^tdRFP^*^/^*^wt^* mice upon MCMV infection, and level of tdRFP expression in these populations.** Left, gating strategies used to define InflDCs in spleen (A) and liver (B). Right, tdRFP expression in these populations isolated from *Xcr1^Cre/wt^;Rosa26^tdRFP^*^/^*^wt^*, *Karma^Cre/wt^;Rosa26^tdRFP^*^/^*^wt^* and *Wt* mice. One mouse of each genotype representative of three is shown. Data are shown for one experiment representative of two.
